# Influence of Pre-treatment Saliva Microbial Diversity and Composition on Nasopharyngeal Carcinoma Prognosis

**DOI:** 10.3389/fcimb.2022.831409

**Published:** 2022-03-22

**Authors:** Yun Du, Ruimei Feng, Ellen T. Chang, Justine W. Debelius, Li Yin, Miao Xu, Tingting Huang, Xiaoying Zhou, Xue Xiao, Yancheng Li, Jian Liao, Yuming Zheng, Guangwu Huang, Hans-Olov Adami, Zhe Zhang, Yonglin Cai, Weimin Ye

**Affiliations:** ^1^ Department of Medical Epidemiology and Biostatistics, Karolinska Institutet, Stockholm, Sweden; ^2^ Department of Epidemiology and Health Statistics and Key Laboratory of Ministry of Education for Gastrointestinal Cancer, Fujian Medical University, Fuzhou, China; ^3^ Exponent, Inc., Center for Health Sciences, Menlo Park, CA, United States; ^4^ Centre for Translational Microbiome Research, Department of Microbiology, Tumor, and Cell Biology, Karolinska Institutet, Solna, Sweden; ^5^ Karolinska Institutet, Science for Life Laboratory, Solna, Sweden; ^6^ State Key Laboratory of Oncology in South China, Collaborative Innovation Center for Cancer Medicine, Guangdong Key Laboratory of Nasopharyngeal Carcinoma Diagnosis and Therapy, Sun Yat-sen University Cancer Center, Guangzhou, China; ^7^ Department of Radiation Oncology, The First Affiliated Hospital of Guangxi Medical University, Nanning, China; ^8^ Radiation Oncology Clinical Medical Research of Guangxi Medical University, Nanning, China; ^9^ Life Science Institute, Guangxi Medical University, Nanning, China; ^10^ Key Laboratory of High-Incidence-Tumor Prevention & Treatment (Guangxi Medical University), Ministry of Education, Nanning, China; ^11^ Department of Otolaryngology-Head & Neck Surgery, First Affiliated Hospital of Guangxi Medical University, Nanning, China; ^12^ Guangxi Health Commission Key Laboratory of Molecular Epidemiology of Nasopharyngeal Carcinoma, Wuzhou Red Cross Hospital, Wuzhou, China; ^13^ Cangwu Institute for Nasopharyngeal Carcinoma Control and Prevention, Wuzhou, China; ^14^ Clinical Effectiveness Research Group, Institute of Health, University of Oslo, Oslo, Norway; ^15^ Department of Epidemiology, Harvard T. H. Chan School of Public Health, Boston, MA, United States

**Keywords:** nasopharyngeal carcinoma, 16S rRNA sequencing, oral microbiome, diversity, prognosis

## Abstract

**Background:**

The human microbiome has been reported to mediate the response to anticancer therapies. However, research about the influence of the oral microbiome on nasopharyngeal carcinoma (NPC) survival is lacking. We aimed to explore the effect of oral microbiota on NPC prognosis.

**Methods:**

Four hundred eighty-two population-based NPC cases in southern China between 2010 and 2013 were followed for survival, and their saliva samples were profiled using 16s rRNA sequencing. We analyzed associations of the oral microbiome diversity with mortality from all causes and NPC.

**Results:**

Within- and between-community diversities of saliva were associated with mortality with an average of 5.29 years follow-up. Lower Faith’s phylogenetic diversity was related to higher all-cause mortality [adjusted hazard ratio (aHR), 1.52 (95% confidence interval (CI), 1.06–2.17)] and NPC-specific mortality [aHR, 1.57 (95% CI, 1.07–2.29)], compared with medium diversity, but higher phylogenetic diversity was not protective. The third principal coordinate (PC3) identified from principal coordinates analysis (PCoA) on Bray–Curtis distance was marginally associated with reduced all-cause mortality [aHR, 0.85 (95% CI, 0.73–1.00)], as was the first principal coordinate (PC1) from PCoA on weighted UniFrac [aHR, 0.86 (95% CI, 0.74–1.00)], but neither was associated with NPC-specific mortality. PC3 from robust principal components analysis was associated with lower all-cause and NPC-specific mortalities, with HRs of 0.72 (95% CI, 0.61–0.85) and 0.71 (95% CI, 0.60–0.85), respectively.

**Conclusions:**

Oral microbiome may be an explanatory factor for NPC prognosis. Lower within-community diversity was associated with higher mortality, and certain measures of between-community diversity were related to mortality. Specifically, candidate bacteria were not related to mortality, suggesting that observed associations may be due to global patterns rather than particular pathogens.

## Introduction

Nasopharyngeal carcinoma (NPC) is a rare cancer in most regions of the world, but high-incidence areas are found in regions of Southeast Asia, North Africa, and the Arctic. The highest rates in the world are reported from southern China due to EBV strain variation and genetic and environmental factors (Plummer et al., 2012; Chang et al., 2021).

Due to the high radio sensitivity of the tumor, the principal treatment radiotherapy yields good survival rates for early-stage disease. The majority of cases, however, presents at an advanced stage, and local failure and distant metastases are frequent (Chen et al., 2019). Studies attempting to identify predictors of NPC prognosis, including genomic (Dai et al., 2016; Tang et al., 2018) and proteomic (You et al., 2019) biomarkers for host and Epstein–Barr virus (EBV)-related factors (Network NCC, 2020), have yielded inconclusive results.

Recent studies have reported that microbial diversity is associated with survival outcomes in patients resected for pancreatic adenocarcinoma (Riquelme et al., 2019), colorectal cancer (Nakatsu et al., 2018), and lung cancer (Peters et al., 2019). The oral ecosystem is related to side effects of radiotherapy, such as severe oral mucositis, which can lead to discontinuation of radiotherapy or chemotherapy (Vera-Llonch et al., 2006). Some bacterial taxa may also be directly associated with response to chemotherapy or radiotherapy (Xu et al., 2014; Geller et al., 2017). We recently reported that changes in the nasopharyngeal microbiome among NPC patients are associated with short-term response to radiotherapy or chemoradiotherapy (Huang et al., 2020). Thus, we hypothesized that global community structure, intracommunity diversity, and taxonomic composition of the oral microbiome might influence NPC prognosis.

To test this hypothesis, we conducted an exploratory population-based study, using saliva bacterial profiles based on 16S rRNA sequencing, to identify associations between oral microbiome characteristics and mortality among NPC patients in southern China (Ye et al., 2017).

## Methods

### Study Population

Population-based NPC cases were enrolled from the Wuzhou region of Guangxi Autonomous Region, China, as one subset of a large case–control study (Ye et al., 2017). Wuzhou was selected for this study due to its relatively high incidence of NPC and low rate of residential mobility. All newly diagnosed cases in Wuzhou were identified through a rapid case ascertainment system involving a network of physicians who diagnosed and/or treated NPC at hospitals in the study area (Ye et al., 2017). Among 792 incident NPC cases identified in Wuzhou between 2010 and 2013, 689 (87%) participated in our cohort (Ye et al., 2017). We excluded 89 cases that rejected providing saliva samples and 58 cases whose saliva DNA were extracted by different method. After excluding three cases that failed library preparation and removing samples with fewer than 1,000 sequences per sample or ambiguous sequencing identifiers, 532 cases had valid sequencing results. We further excluded five cases with an ambiguous diagnosis, one duplicated case, and 32 former smokers [due to the heterogeneity of smoking patterns, as described in detail in our previous paper (Debelius et al., 2020)], leaving 482 (70% of 689 enrolled cases) in the final analysis (**Supplementary Figure S1**).

### Microbiome Assay

At the time of the study interview, unstimulated saliva samples (2–4 ml) were collected into 50-ml falcon tubes with a Tris-EDTA buffer (Quinque et al., 2006) (**Supplementary Material S1**). The median time interval between diagnosis and sample collection was 1 day; 85% of samples were collected within 30 days after diagnosis, most before treatment initiation.

We used 16s rRNA to sequence microbial gene fragments with 341F/805R V3–V4 primers. Protocols for DNA extraction, PCR, and sequencing are described thoroughly in our previous publication (Debelius et al., 2020) and in the supplementary methods (**Supplementary Material S2**). Two blank controls (nuclease-free water) and one single-organism control (*E*. *coli* positive) were included in each batch. Sequencing was performed at Beijing Genome Institute on an Illumina MiSeq using a 2 × 300 bp paired-end strategy.

Sequencing results were processed using QIIME2, as previously described (Debelius et al., 2020). To summarize, sample sequences were demultiplexed, adaptors were trimmed, and paired-end sequences were joined and loaded into QIIME2 (November 2018 release). Subsequently, deblur (v.1.0.4; q2-debur) workflow was applied to denoise and generate amplicon sequences variants (ASVs) with the default parameters after quality filtering (q2 quality filter). Next, a phylogenetic tree was constructed *via* q2 fragment insertion into Greengenes (August 2013) 99% identity tree backbone; ASVs were assigned taxonomy with a naive Bayesian classifier against a pre-trained reference (q2 feature classifier).

ASVs were identified by the first letter of the lowest clearly assigned taxonomic level, the first five letters of their lowest taxonomic assignment, and the first six characters of a MD5 hash of the sequencing.

### Exposure Metrics

Our main exposures were alpha and beta diversity. Alpha diversity was measured by Faith’s phylogenetic diversity (Faith’s PD), observed ASVs, and Shannon diversity index. Observed ASVs provide a measure of microbiome richness, i.e., the number of different sequence variants in the sample. Faith’s PD provides a measure of richness, weighted by the phylogeny (i.e., shared evolutionary history between organisms). Shannon diversity index provides a measure of richness and abundance (i.e., the count of each sequence variant in the sample). Alpha diversity was expressed as a continuous variable or categorized into tertiles (low, medium, and high diversity).

Beta diversity was measured by Bray–Curtis distance, unweighted UniFrac distance, and weighted UniFrac distance using q2 diversity in QIIME2 (Bolyen et al., 2019) after being rarefied to 6,500 sequences. Bray–Curtis considers dissimilarities on relative abundance; weighted UniFrac focuses on relative abundance and phylogeny, with emphasis on abundant microbiomes; and unweighted UniFrac considers presence/absence and phylogeny, with emphasis on rarer organisms.

### Outcome

All-cause mortality and NPC-specific mortality were modeled as outcomes. All cases were followed up for vital status, date of death, and cause of death as of December 31, 2018 through the linkages to Wuzhou Cancer Registry, the Total Population Registry, and the Chinese Centres for Disease Control and Prevention, and by conducting in-person visits and telephone calls with village doctors and contacting local funeral parlous. We verified any ambiguous underlying causes of death by obtaining medical records from hospitals and village doctors. Among the deceased cases, all were classified as having a known cause of death except for one case, for whom we assumed that the cause of death was NPC.

### Covariates

Covariates of all NPC cases, including cancer stage, treatment regimen, body mass index (BMI) before treatment, radiotherapy technique, and nasopharyngeal radiation dose, were extracted from medical charts in 15 hospitals in Guangdong and Guangxi Provinces. Two oncologists assisted by five medical students reviewed the medical records. Cancer stage was re-classified according to the 7th AJCC version (Edge and Compton, 2010) by re-examining imaging reports. One senior oncologist checked a random sample of reports to confirm accuracy.

Potential confounders considered were tobacco use (current, former, or never) (Ouyang et al., 2013; Wu et al., 2016; Spakowicz et al., 2020), missing and filled teeth (classified as the sum of missing and filled teeth after age 20 years: 0, 1, 2, 3–5, or 6+) (Belstrom et al., 2018; Cetindag et al., 2019), teeth brushing frequency (≤1/day and ≥2/day), BMI (<18.5, 18.5–22.9, 23.0–27.5, or >27.5 kg/m^2^) (Lin et al., 2015; Maruvada et al., 2017), and alcohol use (never, former, or current) (Capurso and Lahner, 2017; Chen et al., 2016). We also included residential region (He et al., 2018) and saliva sampling season (Amato et al., 2015) as covariates, since they might affect the oral microbiome, and we included cancer stage, treatment regimen (Network NCC, 2020), education, radiotherapy technique, calendar year of diagnosis, radiotherapy dose, and radiotherapy technique as potential predictors of mortality.

## Statistical Analysis

Univariate associations with all-cause mortality (i.e., overall survival) and NPC-specific mortality (i.e., disease-specific survival) were visualized using Kaplan–Meier curves.

We used univariate Cox proportional hazards regression, with years since diagnosis as the time scale, to evaluate associations between covariates and all-cause or NPC-specific mortality. Covariates with *p <*0.05 were included in multivariate Cox models. Although radiotherapy dose and radiotherapy technique were significant mortality predictors, they were excluded due to high collinearity with other included covariates, resulting in severe variance inflation.

All Cox models took time since diagnosis as underlying timescale. We tested the proportional hazards assumption based on Schoenfeld’s residuals and found no violations. We used likelihood ratio tests to test for interactions. All reported *p*-values are two-sided. We used SAS 9.4 for data management and R 4.0.3 for statistical analysis.

### Alpha Diversity Analysis

The Kruskal–Wallis rank-sum test was used to explore contributors of alpha diversity after z-normalization. *p* < 0.05 was considered the threshold inclusion in multivariate Cox proportional hazards regression models together with significant explanatory mortality predictors. We included age at diagnosis, sex, tobacco use, BMI before treatment, cancer stage, treatment regimen, alcohol use, the number of missing or filled teeth, sequence running number, residential community, and saliva sampling season (**Supplementary Table S1**). Stratified analyses were conducted by age at diagnosis.

### Beta Diversity Analysis

Beta diversity was compared using Adonis *via* the R vegan package (version 2.5.7), adjusted for age, sex, and sequencing running number, with 999 permutations. Covariates with false discovery rate (FDR)-adjusted *p <*0.05 were included in multivariate Cox models together with mortality predictors. It included age at diagnosis, sex, sequencing running number, tobacco use, diagnosis calendar year, the number of missing or filled tooth, cancer stage, treatment regimen, saliva sampling season, BMI before treatments, alcohol use, diagnosis calendar year, and residential community, with or without Faith’s PD (**Supplementary Figure S2**).

Principal coordinates analysis (PCoA) was visualized using Emperor in QIIME2. We took the top three principal coordinates (PCs), corresponding to microbiome pattern, as covariates (after z-normalization) for inclusion in adjusted Cox regression models and tested the three PCs jointly by using likelihood ratio tests to compare nested models with and without the PCs (Plantinga et al., 2017).

### Robust Aitchison Principal-Component Analysis

To account for the sparse compositional nature of microbiome data and the large proportion of zero values (Morton et al., 2017), we applied Aitchison principal-component analysis (RPCA) to describe and visualize beta diversity. RPCA provides a solution to managing the zero-inflation problem *via* matrix completion while preserving feature abundance information to enable identification of taxa that drive the differences among sample groups. The two main procedures in RPCA are transformation (i.e., robust centered log ratio transformation of feature absolute abundance to approximate a normal distribution) and matrix completion (i.e., treating all zero values as missing and building a model to handle the missing data using matrix completion) (Martino et al., 2019). The RPCA, generated using *DEICODE* in QIIME2, reflects the evenness of community (Martino et al., 2019) (code provided in **Supplementary Material S3**). The RPCA metrics and biplots were visualized using Emperor in QIIME2, importing the raw and unrarefied feature tables. Sample loading of PCs as candidates of beta diversity evenness was applied in Cox models as a continuous or ordinal (tertiled) variable.

To assess the relationship between ASV abundance and mortality, we used multivariate Cox models (adjusted for the same covariates as in the beta diversity analysis) to test selected ASVs. Because multiple differential abundance analysis might result in high false-positive rates (Hawinkel et al., 2019), we selected only the ASVs with top 10 and bottom 10 feature loadings of PC3 from RPCA. Meanwhile, the additive log ratio transformation of relative abundance was applied, using the top 10 ASVs as a reference frame to reveal the compositional nature of microbiome data (Morton et al., 2019). FDR-adjusted *p-*values were used.

### Sensitivity Analysis

To confirm the robustness of our results, we performed three sets of sensitivity analyses: first, testing alpha diversity as a continuous variable; second, including 32 former smokers in both the alpha and beta diversity analysis; and third, excluding 55 cases whose saliva samples were collected during or after treatment.

## Results

### Characteristics of the Patients

There were 482 NPC patients included in the analysis (**Table 1**). Their mean age at diagnosis was 48 years, 71% were male, and 93% were diagnosed at stage III or IV. In terms of NPC risk factors, 78% had <9 years of education, 66% brushed their teeth no more than once per day, and 52% were current smokers. Mean (± SD) follow-up time was 5.29 (± 2.07) years; one case was lost to follow-up at 51 months.

**Table 1:** Characteristics of NPC cases and univariate associations between covariates and mortality.

Characteristics	Total n(%)^a^	Deaths n(%)^a^	All-cause HRs (95%CI)	Deaths from NPC n(%)^a^	NPC-specific HRs (95%CI)
**Number of cases**	482 (100.0)	210 (43.6)		181 (37.6)	
**Mean follow-up yeas (SD)**	5.29 (2.07)				
**Mean age at cancer diagnosis (SD)**	48.45 (10.55)		**1.03 (1.01,1.04)**		**1.02 (1.00,1.03)**
**Sex**					
Male	342 (71.0)	165 (78.6)	ref	144 (79.6)	ref
Female	140 (29.0)	45 (21.4)	**0.58 (0.42,0.81)**	37 (20.4)	**0.55 (0.38,0.79)**
**Residential community**					
Wuzhou	108 (22.4)	41 (19.5)	ref	36 (19.9)	ref
Cangwu	115 (23.9)	51 (24.3)	1.26 (0.83,1.89)	46 (25.4)	1.29 (0.83,1.99)
Cenxi	165 (34.2)	79 (37.6)	1.45 (0.99,2.11)	63 (34.8)	1.31 (0.87,1.97)
Tengxian	94 (19.5)	39 (18.6)	1.17 (0.76,1.82)	36 (19.9)	1.23 (0.78,1.96)
**Educational attainment**					
≤ 6 years	204 (42.3)	95 (45.2)	ref	75 (41.4)	ref
7-9 years	170 (35.3)	77 (36.7)	0.96 (0.71,1.30)	70 (38.7)	1.11 (0.80,1.54)
≥ 10 years	108 (22.4)	38 (18.1)	0.72 (0.49,1.04)	36 (19.9)	0.86 (0.58,1.28)
**Tobacco use**					
Never	230 (47.7)	83 (39.5)	ref	67 (37.0)	ref
Current	252 (52.3)	127 (60.5)	**1.56 (1.18,2.06)**	114 (63.0)	**1.73 (1.28,2.34)**
**Diagnosis calendar year**					
2011	224 (46.5)	115 (54.8)	ref	96 (53.0)	ref
2012	149 (30.9)	55 (26.2)	0.75 (0.54,1.04)	53 (29.3)	0.87 (0.62,1.23)
2013	109 (22.6)	40 (19.0)	0.88 (0.61,1.26)	32 (17.7)	0.83 (0.55,1.25)
**Season of saliva sampling**					
Winter	114 (23.7)	51 (24.3)	ref	43 (23.8)	ref
Spring	141 (29.3)	60 (28.6)	0.98 (0.67,1.42)	50 (27.6)	0.97 (0.64,1.46)
Summer	101 (21.0)	45 (21.4)	1.03 (0.69,1.54)	40 (22.1)	1.09 (0.71,1.68)
Autumn	126 (26.1)	54 (25.7)	0.99 (0.67,1.45)	48 (26.5)	1.04 (0.69,1.58)
**Tooth brushing frequency**					
≤ 1/day	316 (65.6)	140 (66.7)	ref	119 (65.7)	ref
≥ 2/day	166 (34.4)	70 (33.3)	0.89 (0.67,1.18)	62 (34.3)	0.93 (0.68,1.26)
**Missing or filled teeth**					
0	209 (43.4)	77 (36.7)	ref	70 (38.7)	ref
1	61 (12.7)	25 (11.9)	1.15 (0.74,1.81)	22 (12.2)	1.12 (0.69,1.80)
2	52 (10.8)	21 (10.0)	1.13 (0.70,1.83)	16 (8.8)	0.95 (0.55,1.63)
3-5	79 (16.4)	39 (18.6)	**1.48 (1.00,2.17)**	36 (19.9)	**1.49 (1.00,2.23)**
6+	81 (16.8)	48 (22.9)	**1.87 (1.31,2.69)**	37 (20.4)	**1.58 (1.06,2.35)**
**Cancer stage**					
I-II	35 (7.3)	5 (2.4)	ref	4 (2.2)	ref
III	209 (43.4)	65 (31.0)	**2.51 (1.01,6.23)**	54 (29.8)	2.60 (0.94,7.19)
IV	238 (49.4)	140 (66.7)	**6.06 (2.48,14.81)**	123 (68.0)	**6.61 (2.44,17.92)**
**Treatment regimen**					
CCRT	250 (51.9)	103 (49.0)	ref	88 (48.6)	ref
CCRT+ICT/ACT	150 (31.1)	66 (31.4)	1.14 (0.84,1.56)	60 (33.1)	1.21 (0.87,1.69)
RT only	60 (12.4)	28 (13.3)	1.22 (0.80,1.85)	20 (11.0)	1.02 (0.63,1.66)
No RT	22 (4.6)	13 (6.2)	**2.01 (1.12,3.57)**	13 (7.2)	**2.33 (1.30,4.17)**
**BMI before treatment**					
Normal Weight	257 (53.3)	130 (61.9)	ref	114 (63.0)	ref
Underweight	62 (12.9)	26 (12.4)	0.78 (0.51,1.19)	23 (12.7)	0.79 (0.50,1.24)
Overweight	93 (19.3)	36 (17.1)	**0.65 (0.45,0.95)**	29 (16.0)	**0.60 (0.40,0.90)**
Obese	70 (14.5)	18 (8.6)	**0.43 (0.26,0.70)**	15 (8.3)	**0.41 (0.24,0.70)**
**History of alcohol use**					
Never	330 (68.5)	134 (63.8)	ref	114 (63.0)	ref
Former	20 (4.1)	13 (6.2)	**1.97 (1.11,3.48)**	10 (5.5)	1.77 (0.93,3.39)
Current	132 (27.4)	63 (30.0)	1.24 (0.92,1.68)	57 (31.5)	1.32 (0.96,1.82)
**Radiotherapy technique**					
No radiotherapy	22 (4.6)	13 (6.2)	ref	13 (7.2)	ref
2DRT/3DRT	266 (55.2)	141 (67.1)	0.67 (0.38,1.19)	119 (65.7)	0.57 (0.32,1.02)
IMRT	194 (40.2)	56 (26.7)	**0.36 (0.19,0.65)**	49 (27.1)	**0.31 (0.17,0.58)**
**Nasopharyngeal radiation dose**					
< 70 Gy	144 (30.1)	41 (19.8)	ref	38 (21.2)	ref
≥ 70 Gy	312 (65.3)	153 (73.9)	**1.73 (1.22,2.44)**	128 (71.5)	**1.56 (1.09,2.24)**
No radiotherapy	22 (4.6)	13 (6.3)	**2.83 (1.52,5.29)**	13 (7.3)	**3.03 (1.61,5.69)**

HRs, hazard ratios; SD, standard deviation; BMI, body mass index; CCRT, concurrent chemoradiotherapy; ICT, induction chemotherapy; ACT, adjuvant chemotherapy; RT, radiotherapy; IMRT, intensity-modulated radiation therapy; 2DRT, conventional 2D radiotherapy; 3DRT, conventional 3D radiotherapy.

a Percentages may not be 100 because of rounding. The statistically significant hazards ratios were in bold.

### Low Alpha Diversity Was Associated With Poorer Overall Survival And Disease-Specific Survival

**Figure 1:**
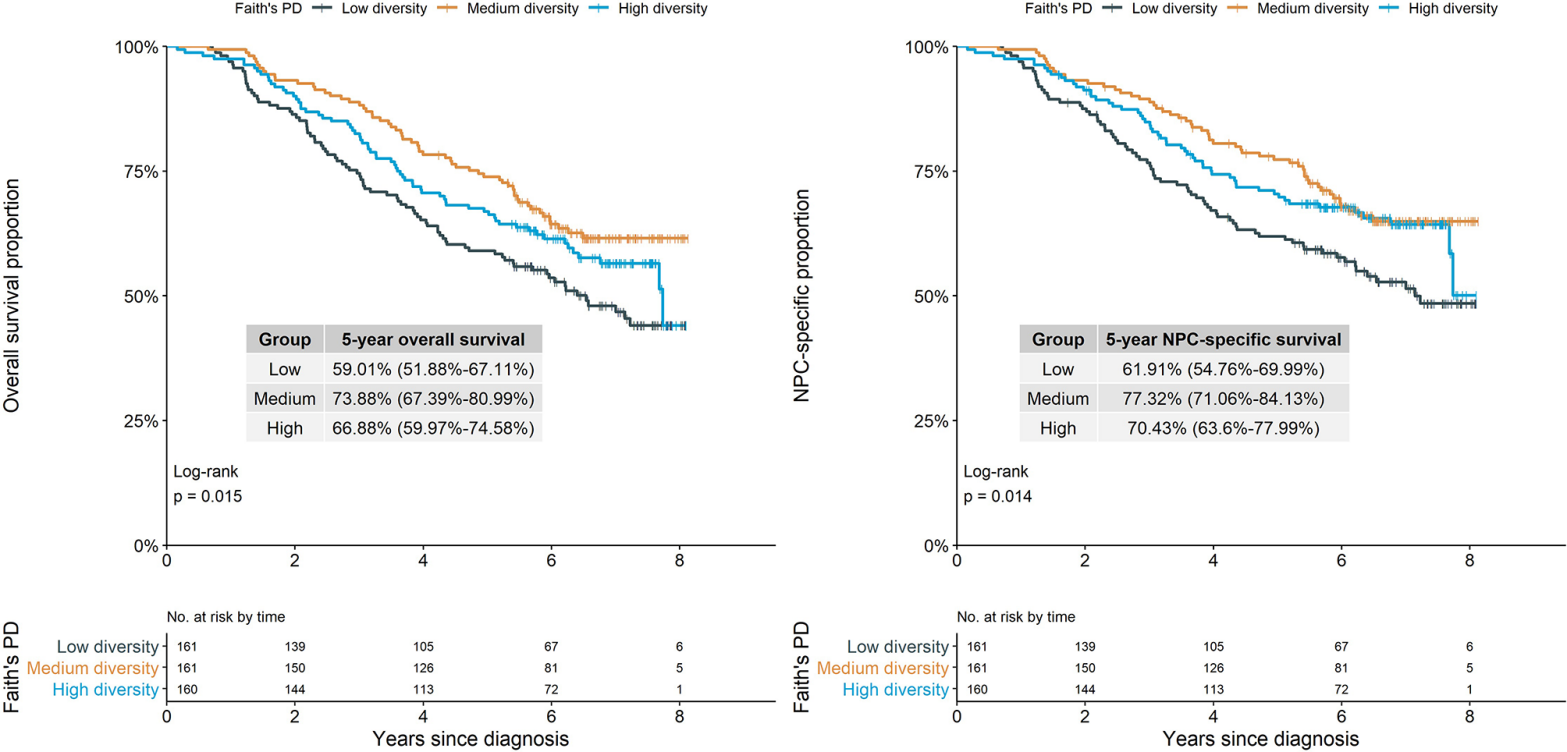
Survival proportion of NPC cases by alpha diversity (Faith’s phylogenetic diversity). Log-rank test showed different Faith’s PD groups had significantly different overall survival and NPC-specific survival proportion (*p*=0.015 and *p*=0.014). NPC, nasopharyngeal carcinoma; Faith’s PD, Faith’s phylogenetic diversity.

Overall survival and NPC-specific survival throughout the study period were considerably and consistently poorest among patients with relatively low Faith’s PD, based on Kaplan–Meier curves (**Figure 1**).

Compared with the medium diversity group based on Faith’s PD, patients with lower Faith’s PD (i.e., lower within-community richness and phylogenetic diversity) had significantly higher all-cause and NPC-specific mortality in both crude and multivariate-adjusted models [adjusted HRs: 1.52 (95% CI, 1.06–2.17) and 1.57 (95% CI, 1.07–2.29)] (**Table 2**). Observed ASVs had a similar pattern of association with all-cause and NPC-specific mortality, although associations were statistically and marginally nonsignificant, whereas Shannon diversity was not associated with either outcome (**Table 2**; **Supplementary Figure S3**).

**Table 2:** Hazard ratios (HRs) for mortality of NPC cases in relation to alpha diversity, Cox regression models.

			All-cause HRs (95%CI)			NPC-specific HRs (95%CI)	
Alpha diversity	Cases (n=482)	Deaths (n=210)	Crude	Adjusted^a^	Deaths from NPC (n=181)	Crude	Adjusted^a^
**Faith’s PD**							
Low diversity	161	83	**1.62 (1.16,2.27)**	**1.52 (1.06,2.17)**	74	**1.64 (1.15,2.33)**	**1.57 (1.07,2.29)**
Medium diversity	161	59	ref	ref	52	ref	ref
High diversity	160	68	1.24 (0.88,1.76)	1.18 (0.82,1.72)	55	1.14 (0.78,1.66)	1.10 (0.73,1.64)
**Observed ASVs**							
Low diversity	161	77	**1.44 (1.03,2.02)**	**1.45 (1.01,2.10)**	68	**1.47 (1.02,2.10)**	1.44 (0.97,2.12)
Medium diversity	161	61	ref	ref	53	ref	ref
High diversity	160	72	1.30 (0.92,1.83)	1.27 (0.88,1.84)	60	1.25 (0.86,1.80)	1.24 (0.83,1.83)
**Shannon**							
Low diversity	161	73	1.05 (0.75,1.45)	1.07 (0.75,1.52)	65	1.08 (0.76,1.53)	1.14 (0.78,1.66)
Medium diversity	161	71	ref	ref	61	ref	ref
High diversity	160	66	0.94 (0.67,1.31)	0.96 (0.68,1.36)	55	0.91 (0.63,1.31)	0.94 (0.64,1.37)

Faith’s PD, Faith’s phylogenetic diversity.

a HRs were adjusted for age, sex, smoking history, BMI before treatment, cancer stage, treatment pattern, alcohol consumption, the number of missing or filled teeth, sequence running number, residential community and season of saliva sampling. The statistically significant hazards ratios were in bold.

In subgroup analysis by age, associations with Faith’s PD and observed ASVs appeared to be stronger among cases of older age (>50 years) vs. younger cases (**Supplementary Figure S4**); however, interactions were statistically nonsignificant.

### Beta Diversity Was Associated With Overall Survival

Because beta diversity was affected by alpha diversity (Debelius et al., 2020), we also controlled for alpha diversity in multivariate models for beta diversity and mortality. Comparing nested models (**Supplementary Table S2**) with and without PCs from PCoA, nested models of PC3 (Bray–Curtis distance) and PC1 (weighted UniFrac distance) were at least marginally significantly associated with all-cause mortality but not with NPC-specific mortality. The adjusted HR for all-cause mortality in association with normalized PC3 from Bray–Curtis distance was 0.85 (95% CI, 0.73–1.00, *p <* 0.05) and that for PC1 from weighted UniFrac distance was 0.86 (95% CI, 0.74–1.00, *p* > 0.05) (**Supplementary Figure S5**). The other two PCs from Bray–Curtis distance and the other two PCs from weighted UniFrac, however, were not significantly associated with all-cause mortality, and none were significantly associated with NPC-specific mortality.

### Robust Aitchison Principal-Component Analysis

**Figure 2:**
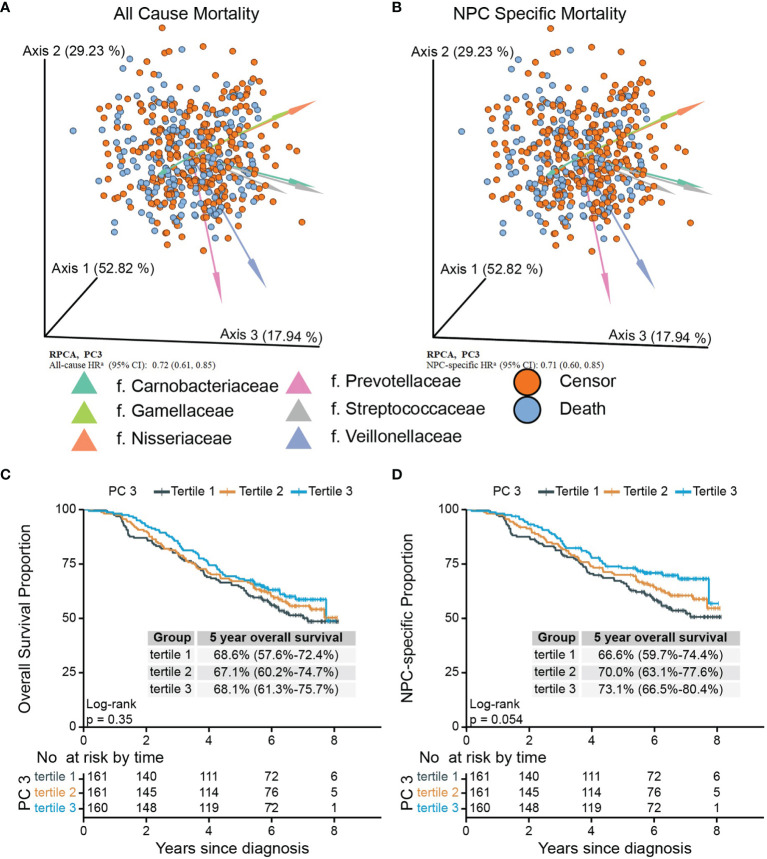
Biplots of RPCA by survival status **(A)** and NPC-specific survival status **(B)** and Kaplan–Meier curves of overall **(C)** and NPC-specific survival **(D)** proportion between tertiled PC3 groups generated from RPCA. PC3 of RPCA were significant mortality predictor **(A, B)** in Cox model with all-cause HR of 0.72 (95% CI, 0.61–0.85) and NPC-specific HR of 0.71 (95% CI, 0.60–0.85). Arrows in **(A)** and **(B)** were top 8 taxa influencing the principal component axis. Axis1, axis2, and axis3 were equal to PC1, PC2, and PC3. The axes were labeled with the variation proportion that PCs explain. Sample loadings PC3 were z-normalized in Cox models. ^a^HRs were adjusted for age at diagnosis, sex, sequencing running number, tobacco use, the number of missing or filled tooth, cancer stage, treatment pattern, saliva sampling season, BMI before treatments, alcohol use, diagnosis calendar year, and residential community and Faith’s PD. PC3 were z-normalized. RPCA, robust Aitchison principal-component analysis; NPC, nasopharyngeal carcinoma; Faith’s PD, Faith’s phylogenetic diversity.

Given that Bray–Curtis and weighted UniFrac reflect relative abundance, we applied RPCA, which takes abundance into account, to derive additional beta diversity metrics. Normalized PC3 was significantly associated with both all-cause mortality [adjusted HR, 0.72 (95% CI, 0.61–0.85)] and NPC-specific mortality [adjusted HR, 0.71 (95% CI, 0.60-0.85)] (**Figure 2A, B**; **Supplementary Table S3**), but PC1 and PC2 from RPCA were not significantly associated with either outcome. To confirm the robustness of the associations with these PCs, we categorized them into tertiles, drew Kaplan–Meier curves, and constructed multivariate Cox models, which shows that tertile 3 vs. tertile 1 of PC3 had 47% lower all-cause mortality and 51% lower NPC-specific mortality (**Figure 2C, D**), whereas associations with PC1 and PC2 remained statistically nonsignificant after adjustment (**Table 3**; **Supplementary Figure S6**).

**Table 3:** Hazard ratios (HRs) for mortality of NPC cases in relation to tertiled PCs from RPCA, Cox regression models.

			All-cause HRs			NPC-specific HRs	
PCs^a^	Cases (n=482)	Deaths (n=210)	Crude	Adjusted^a^	Deaths of NP (n=181)	Crude	Adjusted^b^
**PC1**							
tertile 1	161	63	ref	ref	51	ref	ref
tertile 2	161	81	**1.44 (1.04,2.00)**	1.21 (0.84,1.74)	73	**1.60 (1.12,2.29)**	1.31 (0.89,1.94)
tertile 3	160	66	1.13 (0.80,1.59)	1.30 (0.88,1.93)	57	1.20 (0.82,1.75)	1.32 (0.86,2.02)
**PC2**							
tertile 1	161	69	ref	ref	64	ref	ref
tertile 2	161	75	1.15 (0.83,1.60)	1.07 (0.75,1.54)	61	1.01 (0.71,1.43)	1.01 (0.68,1.48)
tertile 3	160	66	0.99 (0.71,1.39)	1.14 (0.78,1.67)	56	0.90 (0.63,1.29)	1.02 (0.68,1.52)
**PC3**							
tertile 1	161	77	ref	ref	72	ref	ref
tertile 2	161	70	0.88 (0.64,1.22)	**0.66 (0.46,0.96)**	61	0.82 (0.59,1.16)	**0.68 (0.46,1.00)**
tertile 3	160	63	0.78 (0.56,1.09)	**0.53 (0.36,0.80)**	48	**0.64 (0.44,0.92)**	**0.49 (0.32,0.76)**

RPCA, robust Aitchison principal-component analysis; PC, principle component.

a Sample loading of PCs were grouped into three tertiles.

b Adjusted for age at diagnosis, sex, sequencing running number, tobacco use, the number of missing or filled tooth, cancer stage, BMI before treatments, alcohol use, diagnosis calendar year, treatment pattern, saliva sampling season, residential community and Faith’s phylogenetic diversity. The statistically significant hazards ratios were in bold.

We tested for effect modification by performing stratified analysis by age at diagnosis and found stronger associations between tertile PC3 and all-cause and NPC-specific mortality for cases older than 50 years (**Supplementary Figure S4**).

To evaluate whether between-community features are associated with mortality in NPC patients, we tested the top and bottom 10 ASVs with the highest and lowest feature loadings of PC3 from RPCA (**Supplementary Figure S7** **;** **Supplementary Table S4**). None were significantly associated with all-cause or NPC mortality, except for a single modest association between a *Streptococcus* ASV (gStrep.05312a) and all-cause mortality that was nonsignificant after FDR adjustment (*p*=0.018 and FDR=0.368).

### Sensitivity Analysis

When alpha diversity was classified as a continuous variable, higher Faith’s PD, but not the other two measures of alpha diversity, was significantly associated with lower NPC-specific mortality (HR, 0.85; 95% CI, 0.73–1.00), but not all-cause mortality. When we included 32 former smokers or excluded 55 cases with saliva samples during or after treatment in the analysis, results were not meaningfully changed (**Other Supporting Materials**).

## Discussion

The goal of this study was to explore whether the oral microbiome affects mortality among NPC patients in an endemic area. Our results showed that some measures of lower enriched and phylogenetic within-community diversity were related to higher overall and disease-specific mortality, and some measures of between-community diversity and composition were associated with overall mortality. However, results were not consistent, as several measures of alpha and beta diversities were not associated with either overall or NPC-specific mortality.

It is widely believed that a diverse and balanced microbiome plays an essential role in human oral mucosal immune function. The normal commensal microbiota can protect hosts from colonization by exogenous pathogens and overgrowth by indigenous pathobionts. An imbalance between altered commensal microbiota and immune regulation could increase the risk of developing and exacerbating disease (Tomkovich and Jobin, 2016). Interestingly, our stratified results suggest that the impact of diversity on mortality may be more pronounced among elder patients. Previous research reported that older patients with less richness diversity are more prone to treatment complications and lower immunological adaptability (Zawadzki et al., 2017).

Few studies have looked at the relationship between microbiome and long-term cancer prognosis, and to our knowledge, none of these evaluated NPC. Our previous study (Huang et al., 2021) investigated the association between the nasopharyngeal microbiome and short-term response to chemotherapy and radiotherapy, with results suggesting differences in microbial diversity between late and early NPC responders. Hou et al. (2018) found that two microbe candidate taxa in the retropharyngeal wall were significantly related to the progression of oral mucositis, which is a common adverse effect of radiotherapy; this complication could result in discontinuation of chemotherapy or radiotherapy and malnutrition, leading to poorer prognosis among NPC patients. Other studies (Zhu et al., 2017) have reported that as oral mucosal lesions progressed from mild to severe mucositis, their bacterial UniFrac distances from healthy controls increased. Previous results (Xu et al., 2014) also indicated that oral or nasopharyngeal microbes may influence the response to therapy and side effects of radiotherapy among NPC cases. Our findings augment these prior results by suggesting that saliva microbiome diversity might have an important influence on long-term prognosis.

Results from microbiome-based survival studies of other cancers may also be relevant for comparison. Our finding was in line with results showing improved recurrence- and disease-free survival in association with higher richness and evenness of diversity in normal tissue from non-small cell lung cancer patients (Peters et al., 2019). Another study found longer overall survival in association with higher alpha diversity in the tumor tissue from pancreatic adenocarcinoma patients (Riquelme et al., 2019). Among 55 cervical cancer cases, Sims et al. (2020) found that Shannon diversity index for the baseline fecal microbiome was an independent predictor of overall survival and relapse-free survival after chemoradiation treatment. Finally, a meta-analysis (Yang et al., 2020) revealed that antibiotic administration, which decreases microbiome richness, was correlated with poorer overall survival in solid cancer patients, although this observed association could be biased due to confounding by indication (i.e., underlying infection).

Our results showed that the association between mortality and UniFrac diminished after adjustment for alpha diversity. These results suggest that the association of microbiome richness with mortality is more likely to be driven by within-community diversity, whereas the association with between-community diversity is driven mainly by the evenness of beta diversity. Although some previous studies of other cancers (Mitsuhashi et al., 2015; Yamamura et al., 2016; Yamamura et al., 2019) identified certain candidate microbes as predictors of cancer mortality, we found no prominent associations with specific organism. Our results, therefore, suggest that future research focus on community level oral dysbiosis, rather than focusing on interventions with specific organisms.

The strengths of our study include its prospective design, making it the first long-term prospective investigation of the relationship between the oral microbiome and NPC prognosis. Second, the cases are population-based, making results generalizable to NPC in endemic Guangxi Autonomous Region in southern China. Third, by using complementary metrics and broad views of community, we were better positioned to characterize true relationship between microbes and outcomes. Fourth, we had low loss to follow-up (0.2%), thereby minimizing the risk of selection bias. Finally, we collected extensive information on potential confounders, enabling us to adjust for other known and potential prognostic factors.

Limitations include the exploratory nature of the analysis, with numerous statistical tests and the potential for false-positive significant findings. Second, we lacked information on the use of antibiotics and anti-inflammatory therapy before sample collection, which could affect measures of microbiome diversity and survival outcomes. Previous work has suggested that the oral microbiome is more robust to antibiotics than the fecal microbiome (Zaura et al., 2015); however, more research is needed in this area to fully understand the dynamics of the relationship and for full extrapolation to our work. Third, there were 55 cases whose saliva samples were collected during or after treatment. We used the interview date as proxy of sampling date, and in practice, sampling date was 1 or 2 days before interview date. To check the influence of this limitation, we did the sensitivity analysis excluding these 55 cases. Forty-nine of these 55 samples were collected more than 200 days after treatment started (**Figure A in Other Supporting Materials**). The sensitivity analysis’ results remained largely unchanged. Moreover, we have not known the survival outcome when we collected samples and did the sequence of microbiome. The bias in sampling time point was undifferentiated and only drove the results to null direction. Fourth, we did not get the nasopharyngeal samples considering the feasibility of sampling in a population-based context. Finally, our results may not be generalizable outside of southern China, given that the oral microbiome community is strongly determined by dietary pattern and geographic region. More research is needed to assess the generalizability of these results to other populations.

In summary, our study revealed that some measures of oral microbiome diversity are associated with long-term mortality among NPC patients. In particular, lower within-community diversity was associated with poorer mortality, especially among elder cases. Underlying mechanisms, especially the role of immune status, could reveal ways to ameliorate the generally poor prognosis of advanced NPC. We tended to believe that microbiome was the risk factor because most samples were collected before treatment, and we have adjusted lots of confounders and also performed sensitivity analysis, which can increase the evidence of causality. We acknowledge the limitations and lack of external validation and cannot demonstrate directly and strongly that oral microbiome could be biomarkers. However, our results might inspire new ideas on microbiome. We will further extend and validate our results in the future.

## Data Availability Statement

The datasets presented in this study can be found in online repositories. The names of the repository/repositories and accession number(s) can be found below: https://www.ebi.ac.uk/ena, PRJEB37445.

## Ethics Statement

The studies involving human participants were reviewed and approved by Karolinska Institutet, Wuzhou Red Cross Hospital. The patients/participants provided their written informed consent to participate in this study.

## Author Contributions

The study was raised by WY, H-OA, GH and YZ, ZZ, YC, YD, RF, JD, LY, EC, WY, ZZ and YC refined the design. LL, YC, YL and YZ contributed to sample collection and management. TH, XZ and XX were responsible for the lab work. YD, RF, MX, YC and ZZ collected follow-up information and reviewed medical records. YD, JD, LY and RF performed the statistical analysis. Bioinformatic analysis was performed by JD and YD. YD wrote and revised the manuscript. EC provided editing. All were supervised and coordinated by WY. All authors contributed to the article and approved the submitted version.

## Funding

The study was funded by the US (National Cancer Institute grant (R01CA115873 to H-O Adami), the Swedish Research Council (2015-02625, 2015-06268, and 2017-05814 to W. Ye), and the National Nature Science Foundation of China (81272983 to Z. Zhang). Y. Du is partly supported by a grant from China Scholar Council (201806380006).

## Conflict of Interest

Author EC was employed by Exponent, Inc., Center for Health Sciences.

The remaining authors declare that the research was conducted in the absence of any commercial or financial relationships that could be construed as a potential conflict of interest.

## Publisher’s Note

All claims expressed in this article are solely those of the authors and do not necessarily represent those of their affiliated organizations, or those of the publisher, the editors and the reviewers. Any product that may be evaluated in this article, or claim that may be made by its manufacturer, is not guaranteed or endorsed by the publisher.

## Abbreviations

NPC, nasopharyngeal carcinoma; EBV, Epstein–Barr virus; ASVs, amplicon sequences variants; Faith’s PD, Faith’s phylogenetic diversity; BMI, Body Mass Index; FDR, false discovery rate; PCoA, principal coordinates analysis; PCs, principal coordinates; RPCA, robust Aitchison principal component analysis.

## References

[B1] Amato K. R. Leigh S. R. Kent A. Mackie R. I. Yeoman C. J. Stumpf R. M.. (2015). The Gut Microbiota Appears to Compensate for Seasonal Diet Variation in the Wild Black Howler Monkey (Alouatta Pigra). Microb. Ecol. 69 (2), 434–443. doi: 10.1007/s00248-014-0554-7 25524570

[B2] Belstrøm D. Sembler-Møller M. L. Grande M. A Kirkby N. Cotton S. L. Paster B. J.. (2018). Impact of Oral Hygiene Discontinuation on Supragingival and Salivary Microbiomes. JDR. Clin. Trans. Res. 3 (1), 57–64. doi: 10.1177/2380084417723625 29662960 PMC5896869

[B3] Bolyen E. Rideout J. R. Dillon M. R. Bokulich N. A. Abnet C. C. Al-Ghalith G. A.. (2019). Reproducible, Interactive, Scalable and Extensible Microbiome Data Science Using QIIME 2. Nat. Biotechnol. 37 (8), 852–857. doi: 10.1038/s41587-019-0209-9 31341288 PMC7015180

[B4] Capurso G. Lahner E. (2017). The Interaction Between Smoking, Alcohol and the Gut Microbiome. Best Pract. Res. Clin. Gastroenterol. 31 (5), 579–588. doi: 10.1016/j.bpg.2017.10.006 29195678

[B5] Cetindağ M. F. Özsavran A. Y. Yalçin B. Çetindağ İ. Ercan K. Türkölmez Ş.. (2019). The Results of Nasopharyngeal Cancer Patients Treated by Simultaneous Integrated Boost Technique and Concomitant Chemotherapy. Turk J. Med. Sci. 49 (2), 558–565. doi: 10.3906/sag-1605-98 30862133 PMC7018215

[B6] Chang E. T. Ye W. Zeng Y. X. Adami H. O. (2021). The Evolving Epidemiology of Nasopharyngeal Carcinoma. Cancer Epidemiol. Biomarkers Prev. 30 (6), 1035–1047. doi: 10.1158/1055-9965.EPI-20-1702 33849968

[B7] Chen Y. P. Chan A. T. C. Le Q. T. Blanchard P. Sun Y. Ma J. (2019). Nasopharyngeal Carcinoma. Lancet (London England). 394 (10192), 64–80. doi: 10.1016/S0140-6736(19)30956-0 31178151

[B8] Chen Y. P. Zhao B. C. Chen C. Lei X. X. Shen L. J. Chen G.. (2016). Alcohol Drinking as an Unfavorable Prognostic Factor for Male Patients With Nasopharyngeal Carcinoma. Sci. Rep. 6, 19290. doi: 10.1038/srep19290 26776301 PMC4725964

[B9] Dai W. Zheng H. Cheung A. K. Lung M. L. (2016). Genetic and Epigenetic Landscape of Nasopharyngeal Carcinoma. Chin. Clin. Oncol. 5 (2), 16. doi: 10.21037/cco.2016.03.06 27121876

[B10] Debelius J. W. Huang T. Cai Y. Ploner A. Barrett D. Zhou X.. (2020). Subspecies Niche Specialization in the Oral Microbiome Is Associated With Nasopharyngeal Carcinoma Risk. mSystems 5 (4). doi: 10.1128/mSystems.00065-20 PMC7343305 32636333

[B11] Edge S. B. Compton C. C. (2010). The American Joint Committee on Cancer: The 7th Edition of the AJCC Cancer Staging Manual and the Future of TNM. Ann. Surg. Oncol. 17 (6), 1471–1474. doi: 10.1245/s10434-010-0985-4 20180029

[B12] Geller L. T. Barzily-Rokni M. Danino T. Jonas O. H. Shental N. Nejman D.. (2017). Potential Role of Intratumor Bacteria in Mediating Tumor Resistance to the Chemotherapeutic Drug Gemcitabine. Science 357 (6356), 1156–1160. doi: 10.1126/SCIENCE.AAH5043 28912244 PMC5727343

[B13] Hawinkel S. Mattiello F. Bijnens L. Thas O. (2019). A Broken Promise: Microbiome Differential Abundance Methods do Not Control the False Discovery Rate. Brief Bioinform. 20 (1), 210–221. doi: 10.1093/bib/bbx104 28968702

[B14] He Y. Wu W. Zheng H. M. Li P. McDonald D. Sheng H. F.. (2018). Regional Variation Limits Applications of Healthy Gut Microbiome Reference Ranges and Disease Models. Nat. Med. 24 (10), 1532–1535. doi: 10.1038/s41591-018-0164-x 30150716

[B15] Hou J. Zheng H. Li P. Liu H. Zhou H. Yang X. (2018). Distinct Shifts in the Oral Microbiota are Associated With the Progression and Aggravation of Mucositis During Radiotherapy. Radiother. Oncol. 129 (1), 44–51. doi: 10.1016/j.radonc.2018.04.023 29735410

[B16] Huang T. Debelius J. W. Ploner A Xiao X. Zhang T. Hu K.. (2021). Radiotherapy-Induced Changes of the Nasopharyngeal Commensal Microbiome in Nasopharyngeal Carcinoma Patients. Int. J. Radiat. Oncol. Biol. Phys. 109 (1), 145–150. doi: 10.1016/J.IJROBP.2020.08.054 32866565

[B17] Lin Y. H. Chang K. P. Lin Y. S. Chang T. S. (2015). Evaluation of Effect of Body Mass Index and Weight Loss on Survival of Patients With Nasopharyngeal Carcinoma Treated With Intensity-Modulated Radiation Therapy. Radiat. Oncol. 10, 136. doi: 10.1186/s13014-015-0443-3 26122711 PMC4486696

[B18] Martino C. Morton J. T. Marotz C. A. Thompson L. R. Tripathi A. Knight R.. (2019). A Novel Sparse Compositional Technique Reveals Microbial Perturbations. mSystems 4 (1), e00016–e00019. doi: 10.1128/mSystems.00016-19 30801021 PMC6372836

[B19] Maruvada P. Leone V. Kaplan L. M. Chang E. B. (2017). The Human Microbiome and Obesity: Moving Beyond Associations. Cell Host Microbe 22 (5), 589–599. doi: 10.1016/j.chom.2017.10.005 29120742

[B20] Mitsuhashi K. Nosho K. Sukawa Y. Matsunaga Y. Ito M. Kurihara H.. (2015). Association of Fusobacterium Species in Pancreatic Cancer Tissues With Molecular Features and Prognosis. Oncotarget 6 (9), 7209–7220. doi: 10.18632/oncotarget.3109 25797243 PMC4466679

[B21] Morton J. T. Marotz C. Washburne A. Silverman J. Zaramela L. S. Edlund A.. (2019). Establishing Microbial Composition Measurement Standards With Reference Frames. Nat. Commun. 10 (1), 2719. doi: 10.1038/s41467-019-10656-5 31222023 PMC6586903

[B22] Morton J. T. Toran L. Edlund A. Metcalf J. L. Lauber C. Knight R. (2017). Uncovering the Horseshoe Effect in Microbial Analyses. mSystems 2 (1), e00166–16. doi: 10.1128/mSystems.00166-16 PMC5320001 28251186

[B23] Nakatsu G. Zhou H. Wu W. K. K. Wong S. H. Coker O. O. Dai Z.. (2018). Alterations in Enteric Virome Are Associated With Colorectal Cancer and Survival Outcomes. Gastroenterology 155 (2), 529–541 e525. doi: 10.1053/j.gastro.2018.04.018 29689266

[B24] Network NCC (2020) Head and Neck Cancers (Version 1.2021). Available at: https://www.nccn.org/professionals/physician_gls/pdf/head-and-neck.pdf (Accessed Nov 9, 2020).

[B25] Yang P. Y. O. Su Z. Mao Y. P. Liang X. X. Liu Q. Deng W.. (2013). Prognostic Impact of Cigarette Smoking on the Survival of Patients With Established Nasopharyngeal Carcinoma. Cancer Epidemiol. Biomarkers Prev. 22 (12), 2285–2294. doi: 10.1158/1055-9965.EPI-13-0546 24252872

[B26] Peters B. A. Hayes R. B. Goparaju C. Reid C. Pass H. I. Ahn J. (2019). The Microbiome in Lung Cancer Tissue and Recurrence-Free Survival. Cancer Epidemiol. Biomarkers Prev. 28 (4), 731–740. doi: 10.1158/1055-9965.EPI-18-0966 30733306 PMC6449216

[B27] Plantinga A. Zhan X. Zhao N. Chen J. Jenq R. R. Wu M. C. (2017). MiRKAT-S: A Community-Level Test of Association Between the Microbiota and Survival Times. Microbiome 5 (1), 17. doi: 10.1186/s40168-017-0239-9 28179014 PMC5299808

[B28] Plummer M. de Martel C. Vignat J. Ferlay J. Bray F. Franceschi S. (2016). Global Burden of Cancers Attributable to Infections in 2012: A Synthetic Analysis. Lancet Glob. Health 4 (9), e609–e616. doi: 10.1016/S2214-109X(16)30143-7 27470177

[B29] Quinque D. Kittler R. Kayser M. Stoneking M. Nasidze I. (2006). Evaluation of Saliva as a Source of Human DNA for Population and Association Studies. Anal. Biochem. 353 (2), 272–277. doi: 10.1016/j.ab.2006.03.021 16620753

[B30] Riquelme E. Zhang Y. Zhang L. Montiel M. Zoltan M. Dong W.. (2019). Tumor Microbiome Diversity and Composition Influence Pancreatic Cancer Outcomes. Cell 178 (4), 795–806.e712. doi: 10.1016/j.cell.2019.07.008 31398337 PMC7288240

[B31] Sims T. T. el Alam M. B. Karpinets T. Dorta-Estremera S. Hegde V. L. Nookala S.. (2020). Gut Microbiome Diversity as an Independent Predictor of Survival in Cervical Cancer Patients Receiving Chemoradiation. J. Clin. Oncol. 38 (15_suppl), 6036–6036. doi: 10.1200/JCO.2020.38.15_suppl.6036 PMC7900251 33619320

[B32] Spakowicz D. Hoyd R. Muniak M. Husain M. Bassett J. S. Wang L.. (2020). Inferring the Role of the Microbiome on Survival in Patients Treated With Immune Checkpoint Inhibitors: Causal Modeling, Timing, and Classes of Concomitant Medications. BMC Cancer 20 (1), 383. doi: 10.1186/s12885-020-06882-6 32375706 PMC7201618

[B33] Tang X. R. Li Y. Q. Liang S. B. Jiang W. Liu F. Ge W. X.. (2018). Development and Validation of a Gene Expression-Based Signature to Predict Distant Metastasis in Locoregionally Advanced Nasopharyngeal Carcinoma: A Retrospective, Multicentre, Cohort Study. Lancet Oncol. 19 (3), 382–393. doi: 10.1016/S1470-2045(18)30080-9 29428165

[B34] Tomkovich S. Jobin C. (2016). Microbiota and Host Immune Responses: A Love-Hate Relationship. Immunology 147 (1), 1–10. doi: 10.1111/imm.12538 26439191 PMC4693877

[B35] Vera-Llonch M. Oster G. Hagiwara M. Sonis S. (2006). Oral Mucositis in Patients Undergoing Radiation Treatment for Head and Neck Carcinoma. Cancer 106 (2), 329–336. doi: 10.1002/cncr.21622 16342066

[B36] Wu J. Peters B. A. Dominianni C. Zhang Y. Pei Z. Yang L.. (2016). Cigarette Smoking and the Oral Microbiome in a Large Study of American Adults. ISME. J. 10 (10), 2435–2446. doi: 10.1038/ismej.2016.37 27015003 PMC5030690

[B37] Xu Y. Teng F. Huang S. Lin Z. Yuan X. Zeng X.. (2014). Changes of Saliva Microbiota in Nasopharyngeal Carcinoma Patients Under Chemoradiation Therapy. Arch. Oral. Biol. 59 (2), 176–186. doi: 10.1016/j.archoralbio.2013.10.011 24370189

[B38] Yamamura K. Baba Y. Nakagawa S. Mima K. Miyake K. Nakamura K.. (2016). Human Microbiome Fusobacterium Nucleatum in Esophageal Cancer Tissue Is Associated With Prognosis. Clin. Cancer Res. 22 (22), 5574–5581. doi: 10.1158/1078-0432.CCR-16-1786 27769987

[B39] Yamamura K. Izumi D. Kandimalla R. Sonohara F. Baba Y. Yoshida N.. (2019). Intratumoral Fusobacterium Nucleatum Levels Predict Therapeutic Response to Neoadjuvant Chemotherapy in Esophageal Squamous Cell Carcinoma. Clin. Cancer Res. 25 (20), 6170–6179. doi: 10.1158/1078-0432.CCR-19-0318 31358543 PMC6801075

[B40] Yang M. Wang Y. Yuan M. Tao M. Kong C. Li H.. (2020). Antibiotic Administration Shortly Before or After Immunotherapy Initiation is Correlated With Poor Prognosis in Solid Cancer Patients: An Up-to-Date Systematic Review and Meta-Analysis. Int. Immunopharmacol. 88, 106876. doi: 10.1016/j.intimp.2020.106876 32799113

[B41] Ye W. Chang E. T. Liu Z. Liu Q. Cai Y. Zhang Z.. (2017). Development of a Population-Based Cancer Case-Control Study in Southern China. Oncotarget 8 (50), 87073–87085. doi: 10.18632/oncotarget.19692 29152064 PMC5675616

[B42] You R. Liu Y. P. Lin M. Huang P. Y. Tang L. Q. Zhang Y. N.. (2019). Relationship of Circulating Tumor Cells and Epstein-Barr Virus DNA to Progression-Free Survival and Overall Survival in Metastatic Nasopharyngeal Carcinoma Patients. Int. J. Cancer. 145 (10), 2873–2883. doi: 10.1002/ijc.32380 31044420

[B43] Zaura E. Brandt B. W. de Mattos M. J. T. Buijs M. J. Caspers M. P. M. Rashid M. U.. (2015). Same Exposure But Two Radically Different Responses to Antibiotics: Resilience of the Salivary Microbiome Versus Long-Term Microbial Shifts in Feces. mBio 6 (6), e01693–e01615. doi: 10.1128/mBio.01693-15 26556275 PMC4659469

[B44] Zawadzki P. J. Perkowski K. Padzik M. Mierzwińska-Nastalska E. Szaflik J. P. Conn D. B.. (2017). Examination of Oral Microbiota Diversity in Adults and Older Adults as an Approach to Prevent Spread of Risk Factors for Human Infections. BioMed. Res. Int. 2017, 8106491. doi: 10.1155/2017/8106491 29082256 PMC5610830

[B45] Zhu X. X. Yang X. J. Chao Y. L. Zheng H. M. Sheng H. F. Liu H. Y.. (2017). The Potential Effect of Oral Microbiota in the Prediction of Mucositis During Radiotherapy for Nasopharyngeal Carcinoma. EBioMedicine 18, 23–31. doi: 10.1016/j.ebiom.2017.02.002 28216066 PMC5405060

