# Supplementary material for: Influence of Pre-treatment Saliva Microbial Diversity and Composition on Nasopharyngeal Carcinoma Prognosis
Source: Front Cell Infect Microbiol. 2022 Mar 22;12:831409. doi: 10.3389/fcimb.2022.831409 (PMC8981580; doi:10.3389/fcimb.2022.831409)
Supplement: Supplementary file 1 [file DataSheet_1.zip › Supplementary_OtherSupportingMaterials/Other_Supporting_materials_frontiers_20220313.docx]

**Other supporting materialss**

# Sensitivity analysis

## Alpha diversity as numeric variables

### Table A

Table A. Hazard ratios (HRs) in relation to alpha diversity (numeric variables) in Cox model for mortality

|  | **All-cause HRs (95%CI)** | | **NPC-specific HRs (95%CI)** | |
| --- | --- | --- | --- | --- |
| **Alpha diversity^*^** | **Crude** | **Adjusted^†^** | **Crude** | **Adjusted^†^** |
| Faith’s PD | 0.90 (0.79,1.04) | 0.89 (0.77,1.04) | 0.86 (0.74,1.00) | 0.85 (0.73,1.00) |
| Observed ASVs | 0.92 (0.80,1.06) | 0.90 (0.78,1.05) | 0.87 (0.75,1.01) | 0.87 (0.74,1.01) |
| Shannon | 0.96 (0.84,1.10) | 0.95 (0.82,1.10) | 0.93 (0.80,1.07) | 0.91 (0.78,1.07) |
| Abbreviations: Faith’s PD, Faith’s phylogenetic diversity. | | | | |
| ^*^Alpha diversity was z-normalized. | | | | |
| ^†^HRs were adjusted for age, sex, smoking history, BMI before treatment, cancer stage, treatment pattern, alcohol consumption, the number of missing or repaired teeth, sequence running number, residential community and season of saliva sampling. | | | | |
|  |  |  |  |  |

## Including former smokers into analysis

### Table B

Table B. Characteristics of NPC cases

| **Characteristics** | **Groups** | **Overall^†^** |
| --- | --- | --- |
| Number of cases |  | 514 |
| Total person-years |  | 2695 |
| Mean follow-up years (SD) |  | 5.24 (2.09) |
| Death event, number (%) | Alive | 285 (55.4) |
|  | Death | 227 (44.2) |
|  | Loss to follow-up | 2 ( 0.4) |
| Causes of death, number (%) | Alive | 285 (55.4) |
|  | Died from NPC | 195 (37.9) |
|  | Died from other causes | 32 ( 6.2) |
|  | Loss to follow-up | 2 ( 0.4) |
| Sex, number (%) | Male | 374 (72.8) |
|  | Female | 140 (27.2) |
| Age at cancer diagnosis, mean (SD) |  | 48.85 (10.50) |
| Residential community, number (%) | Wuzhou | 117 (22.8) |
|  | Cangwu | 122 (23.7) |
|  | Cenxi | 174 (33.9) |
|  | Tengxian | 101 (19.6) |
| Educational attainment, number (%) | ≤ 6 years | 215 (41.8) |
|  | 7-9 years | 183 (35.6) |
|  | ≥ 10 years | 116 (22.6) |
| Tobacco use, number (%) | Never | 230 (44.7) |
|  | Former | 32 ( 6.2) |
|  | Current | 252 (49.0) |
| Diagnosis calendar year, number (%) | 2011 | 235 (45.7) |
|  | 2012 | 163 (31.7) |
|  | 2013 | 116 (22.6) |
| Season of saliva sampling, number (%) | Winter | 121 (23.5) |
|  | Spring | 150 (29.2) |
|  | Summer | 108 (21.0) |
|  | Autumn | 135 (26.3) |
| Tooth brushing frequency, number (%) | ≤ 1/day | 336 (65.4) |
|  | ≥ 2/day | 178 (34.6) |
| Missing or repaired teeth, number (%) | 0 | 224 (43.6) |
|  | 1 | 65 (12.6) |
|  | 2 | 58 (11.3) |
|  | 3-5 | 84 (16.3) |
|  | 6+ | 83 (16.1) |
| Cancer stage, number (%) | I-II | 42 ( 8.2) |
|  | III | 220 (42.8) |
|  | IV | 252 (49.0) |
| Treatment pattern, number (%) | CCRT | 267 (51.9) |
|  | CCRT+ICT/ACT | 160 (31.1) |
|  | RT only | 64 (12.5) |
|  | No RT | 23 ( 4.5) |
| BMI before treatment, number (%) | Normal Weight | 274 (53.3) |
|  | Underweight | 65 (12.6) |
|  | Overweight | 98 (19.1) |
|  | Obese | 77 (15.0) |
| History of alcohol use, number (%) | Never | 348 (67.7) |
|  | Former | 24 ( 4.7) |
|  | Current | 142 (27.6) |
| Abbreviations: SD, standard deviation; BMI, body mass index; CCRT: concurrent chemoradiotherapy; ICT: induction chemotherapy; ACT: adjuvant chemotherapy; RT: radiotherapy | | |
| ^†^Percentages may not be 100 because of rounding. | | |

### Table C

Table C. Hazard ratios (HRs) in relation to alpha diversity in Cox model for all-cause and NPC-specific mortality

|  | | **All-cause** | | | **NPC-specific** | | |
| --- | --- | --- | --- | --- | --- | --- | --- |
| **Alpha diversity** | **Cases   (n=514)** | **Events   (n=227)** | **Crude HRs** | **Adjusted HRs^†^** | **Events   (n=195)** | **Crude HRs** | **Adjusted HRs^†^** |
| **Phylogenetic Diversity** |  |  |  |  |  |  |  |
| Low diversity | 172 | 88 | **1.54 (1.12,2.12)** | **1.44 (1.02,2.04)** | 79 | **1.57 (1.12,2.21)** | **1.48 (1.03,2.15)** |
| Medium diversity | 171 | 65 | ref | ref | 57 | ref | ref |
| High diversity | 171 | 74 | 1.21 (0.87,1.70) | 1.17 (0.82,1.67) | 59 | 1.10 (0.77,1.59) | 1.07 (0.73,1.58) |
| **Observed ASVs** |  |  |  |  |  |  |  |
| Low diversity | 172 | 83 | **1.42 (1.03,1.96)** | **1.47 (1.03,2.09)** | 73 | **1.45 (1.02,2.04)** | 1.44 (0.99,2.11) |
| Medium diversity | 171 | 66 | ref | ref | 57 | ref | ref |
| High diversity | 171 | 78 | 1.29 (0.93,1.80) | 1.35 (0.95,1.92) | 65 | 1.25 (0.87,1.78) | 1.31 (0.89,1.91) |
| **Shannon** |  |  |  |  |  |  |  |
| Low diversity | 172 | 79 | 1.07 (0.78,1.47) | 1.10 (0.79,1.55) | 71 | 1.12 (0.80,1.58) | 1.17 (0.81,1.68) |
| Medium diversity | 171 | 75 | ref | ref | 64 | ref | ref |
| High diversity | 171 | 73 | 0.98 (0.71,1.36) | 1.02 (0.73,1.42) | 60 | 0.95 (0.67,1.35) | 0.97 (0.67,1.40) |
| ^†^HRs were adjusted for age, sex, smoking history, BMI before treatment, cancer stage, treatment pattern, alcohol consumption, the number of missing or repaired teeth, sequence running number, residential community and season of saliva sampling. | | | | | | | |
|  |  |  |  |  |  |  |  |

### Table D

Table D. Hazard ratios (HRs) of tertiled PCs from RPCA on mortality using Cox regression

|  | | | All-cause HRs | |  | NPC-specific HRs | | |  |
| --- | --- | --- | --- | --- | --- | --- | --- | --- | --- |
| PCs | Cases   (n=514) | Deaths   (n=227) | Crude | Adjusted^*^ | Deaths of NPC   (n=195) | | Crude | Adjusted^*^ | |
| **PC1** |  |  |  |  |  | |  |  | |
| tertile 1 | 172 | 73 | ref | ref | 62 | | ref | ref | |
| tertile 2 | 171 | 78 | 1.06 (0.77,1.46) | 0.87 (0.62,1.23) | 68 | | 1.09 (0.77,1.54) | 0.91 (0.63,1.31) | |
| tertile 3 | 171 | 76 | 1.00 (0.73,1.38) | 0.95 (0.66,1.37) | 65 | | 1.01 (0.71,1.43) | 1.00 (0.67,1.47) | |
| **PC2** |  |  |  |  |  | |  |  | |
| tertile 1 | 172 | 78 | ref | ref | 70 | | ref | ref | |
| tertile 2 | 171 | 82 | 1.08 (0.79,1.48) | 0.91 (0.65,1.27) | 69 | | 1.01 (0.73,1.41) | 0.91 (0.64,1.30) | |
| tertile 3 | 171 | 67 | 0.86 (0.62,1.19) | 0.81 (0.56,1.18) | 56 | | 0.80 (0.56,1.13) | 0.77 (0.52,1.16) | |
| **PC3** |  |  |  |  |  | |  |  | |
| tertile 1 | 172 | 88 | ref | ref | 79 | | ref | ref | |
| tertile 2 | 171 | 72 | 0.81 (0.60,1.11) | 0.73 (0.52,1.04) | 63 | | 0.79 (0.57,1.10) | 0.81 (0.56,1.16) | |
| tertile 3 | 171 | 67 | **0.69 (0.50,0.95)** | **0.52 (0.35,0.78)** | **53** | | **0.61 (0.43,0.87)** | **0.53 (0.34,0.81)** | |
| Abbreviation: RPCA, robust Aitchison principal-component analysis. | | | | | | | | |  |
| ^*^Adjusted for age at diagnosis, sex, sequencing running number, tobacco use, the number of missing or repaired tooth, cancer stage, BMI before treatments, alcohol use, diagnosis calendar year, treatment pattern, saliva sampling season, residential community and Faith’s phylogenetic diversity. | | | | | | | | |  |
|  |  |  |  |  |  |  |  |  |  |

## Exclude cases whose saliva samples were collected during or after treatment

### Table E

Table E. Characteristics of NPC cases

| **Characteristics** | **Total   n(%)^†^** | **Deaths   n(%)^†^** | **All-cause HRs   (95%CI)** | **Deaths from NPC   n(%)^†^** | **NPC-specific HRs   (95%CI)** |
| --- | --- | --- | --- | --- | --- |
| **Number of cases** | 427 (100.0) | 193 (45.2) |  | 167 (39.1) |  |
| **Mean follow-up years (SD)** | 5.29 (2.07) |  |  |  |  |
| **Mean age at cancer diagnosis (SD)** | 48.19 (10.80) |  | **1.02 (1.01,1.04)** |  | **1.02 (1.00,1.03)** |
| **Sex** |  |  |  |  |  |
| Male | 302 (70.7) | 150 (77.7) | ref | 132 (79.0) | ref |
| Female | 125 (29.3) | 43 (22.3) | **0.61 (0.44,0.86)** | 35 (21.0) | **0.57 (0.39,0.83)** |
| **Residential community** |  |  |  |  |  |
| Wuzhou | 86 (20.1) | 35 (18.1) | ref | 31 (18.6) | ref |
| Cangwu | 115 (26.9) | 51 (26.4) | 1.18 (0.77,1.81) | 46 (27.5) | 1.20 (0.76,1.89) |
| Cenxi | 150 (35.1) | 73 (37.8) | 1.38 (0.92,2.06) | 58 (34.7) | 1.23 (0.80,1.90) |
| Tengxian | 76 (17.8) | 34 (17.6) | 1.19 (0.75,1.92) | 32 (19.2) | 1.27 (0.77,2.08) |
| **Educational attainment** |  |  |  |  |  |
| ≤ 6 years | 187 (43.8) | 91 (47.2) | ref | 72 (43.1) | ref |
| 7-9 years | 145 (34.0) | 67 (34.7) | 0.93 (0.68,1.28) | 62 (37.1) | 1.09 (0.78,1.54) |
| ≥ 10 years | 95 (22.2) | 35 (18.1) | 0.72 (0.49,1.07) | 33 (19.8) | 0.87 (0.57,1.31) |
| **Tobacco use** |  |  |  |  |  |
| Never | 203 (47.5) | 76 (39.4) | ref | 61 (36.5) | ref |
| Current | 224 (52.5) | 117 (60.6) | **1.55 (1.16,2.07)** | 106 (63.5) | **1.74 (1.27,2.39)** |
| **Diagnosis calendar year** |  |  |  |  |  |
| 2011 | 222 (52.0) | 114 (59.1) | ref | 95 (56.9) | ref |
| 2012 | 125 (29.3) | 50 (25.9) | 0.84 (0.60,1.17) | 48 (28.7) | 0.97 (0.68,1.38) |
| 2013 | 80 (18.7) | 29 (15.0) | 0.87 (0.57,1.31) | 24 (14.4) | 0.85 (0.54,1.34) |
| **Season of saliva sampling** |  |  |  |  |  |
| Winter | 103 (24.1) | 48 (24.9) | ref | 41 (24.6) | ref |
| Spring | 126 (29.5) | 54 (28.0) | 0.95 (0.64,1.40) | 45 (26.9) | 0.93 (0.61,1.42) |
| Summer | 101 (23.7) | 45 (23.3) | 1.00 (0.66,1.50) | 40 (24.0) | 1.04 (0.67,1.62) |
| Autumn | 97 (22.7) | 46 (23.8) | 1.09 (0.73,1.63) | 41 (24.6) | 1.14 (0.74,1.75) |
| **Tooth brushing frequency** |  |  |  |  |  |
| ≤ 1/day | 284 (66.5) | 132 (68.4) | ref | 114 (68.3) | ref |
| ≥ 2/day | 143 (33.5) | 61 (31.6) | 0.85 (0.63,1.15) | 53 (31.7) | 0.86 (0.62,1.19) |
| **Missing or repaired teeth** |  |  |  |  |  |
| 0 | 177 (41.5) | 69 (35.8) | ref | 64 (38.3) | ref |
| 1 | 58 (13.6) | 24 (12.4) | 1.09 (0.68,1.73) | 22 (13.2) | 1.07 (0.66,1.74) |
| 2 | 48 (11.2) | 21 (10.9) | 1.18 (0.72,1.92) | 16 ( 9.6) | 0.97 (0.56,1.67) |
| 3-5 | 74 (17.3) | 38 (19.7) | 1.45 (0.98,2.16) | 35 (21.0) | 1.44 (0.95,2.17) |
| 6+ | 70 (16.4) | 41 (21.2) | **1.71 (1.16,2.52)** | 30 (18.0) | 1.34 (0.87,2.07) |
| **Cancer stage** |  |  |  |  |  |
| I-II | 31 ( 7.3) | 4 ( 2.1) | ref | 3 ( 1.8) | ref |
| III | 182 (42.6) | 59 (30.6) | **2.94 (1.07,8.09)** | 49 (29.3) | **3.24 (1.01,10.42)** |
| IV | 214 (50.1) | 130 (67.4) | **7.08 (2.61,19.17)** | 115 (68.9) | **8.27 (2.62,26.06)** |
| **Treatment pattern** |  |  |  |  |  |
| CCRT | 229 (53.6) | 98 (50.8) | ref | 83 (49.7) | ref |
| CCRT+ICT/ACT | 124 (29.0) | 57 (29.5) | 1.15 (0.83,1.59) | 53 (31.7) | 1.26 (0.89,1.78) |
| RT only | 53 (12.4) | 25 (13.0) | 1.16 (0.75,1.81) | 18 (10.8) | 0.99 (0.59,1.65) |
| No RT | 21 ( 4.9) | 13 ( 6.7) | **2.10 (1.18,3.75)** | 13 ( 7.8) | **2.45 (1.36,4.41)** |
| **BMI before treatments** |  |  |  |  |  |
| Normal Weight | 232 (54.3) | 120 (62.2) | ref | 106 (63.5) | ref |
| Underweight | 56 (13.1) | 25 (13.0) | 0.82 (0.53,1.26) | 22 (13.2) | 0.82 (0.51,1.29) |
| Overweight | 77 (18.0) | 32 (16.6) | **0.68 (0.46,1.00)** | 26 (15.6) | **0.62 (0.40,0.96)** |
| Obese | 62 (14.5) | 16 ( 8.3) | **0.42 (0.25,0.70)** | 13 ( 7.8) | **0.39 (0.22,0.69)** |
| **History of alcohol use** |  |  |  |  |  |
| Never | 294 (68.9) | 123 (63.7) | ref | 105 (62.9) | ref |
| Former | 17 ( 4.0) | 12 ( 6.2) | **2.11 (1.17,3.82)** | 9 ( 5.4) | 1.84 (0.93,3.64) |
| Current | 116 (27.2) | 58 (30.1) | 1.26 (0.92,1.72) | 53 (31.7) | 1.35 (0.97,1.87) |
| **Radiotherapy technique** |  |  |  |  |  |
| No radiotherapy | 21 ( 4.9) | 13 ( 6.7) | ref | 13 ( 7.8) | ref |
| 2DRT/3DRT | 255 (59.7) | 137 (71.0) | 0.63 (0.35,1.11) | 115 (68.9) | **0.53 (0.30,0.94)** |
| IMRT | 151 (35.4) | 43 (22.3) | **0.32 (0.17,0.59)** | 39 (23.4) | **0.29 (0.16,0.55)** |
| **Nasopharyngeal radiation dose** |  |  |  |  |  |
| < 70 Gy | 114 (27.0) | 33 (17.4) | ref | 32 (19.4) | ref |
| ≥ 70Gy | 288 (68.1) | 144 (75.8) | **1.72 (1.18,2.52)** | 120 (72.7) | **1.48 (1.00,2.19)** |
| No radiotherapy | 21 ( 5.0) | 13 ( 6.8) | **3.03 (1.60,5.77)** | 13 ( 7.9) | **3.09 (1.62,5.89)** |
| Abbreviations: HRs, hazard ratios; SD, standard deviation; BMI, body mass index; CCRT, concurrent chemoradiotherapy; ICT, induction chemotherapy; ACT, adjuvant chemotherapy; RT, radiotherapy; IMRT, intensity-modulated radiation therapy. | | | | | |
| ^†^Percentages may not be 100 because of rounding. | | | | | |
|  |  |  |  |  |  |
|  |  |  |  |  |  |

### Table F

Table F. Hazard ratios (HRs) for mortality of NPC cases in relation to alpha diversity, Cox regression models

|  | | | **All-cause HRs (95%CI)** | |  | **NPC-specific HRs (95%CI)** | |
| --- | --- | --- | --- | --- | --- | --- | --- |
| **Alpha diversity** | **Cases   (n=427)** | **Deaths   (n=193)** | **Crude** | **Adjusted^†^** | **Deaths from NPC   (n=167)** | **Crude** | **Adjusted^†^** |
| **Faith PD** |  |  |  |  |  |  |  |
| Low diversity | 143 | 76 | **1.49 (1.06,2.10)** | 1.38 (0.95,2.01) | 67 | **1.44 (1.00,2.07)** | 1.34 (0.91,1.99) |
| Medium diversity | 142 | 57 | ref | ref | 52 | ref | ref |
| High diversity | 142 | 60 | 1.10 (0.77,1.58) | 0.99 (0.67,1.46) | 48 | 0.97 (0.65,1.43) | 0.87 (0.57,1.33) |
| **Observed ASVs** |  |  |  |  |  |  |  |
| Low diversity | 143 | 71 | **1.46 (1.03,2.07)** | **1.48 (1.00,2.17)** | 64 | **1.50 (1.03,2.18)** | 1.48 (0.98,2.22) |
| Medium diversity | 142 | 56 | ref | ref | 49 | ref | ref |
| High diversity | 142 | 66 | 1.32 (0.93,1.89) | 1.29 (0.88,1.90) | 54 | 1.23 (0.84,1.82) | 1.21 (0.80,1.84) |
| **Shannon** |  |  |  |  |  |  |  |
| Low diversity | 143 | 65 | 1.02 (0.73,1.44) | 1.07 (0.74,1.56) | 59 | 1.08 (0.75,1.55) | 1.19 (0.80,1.76) |
| Medium diversity | 142 | 65 | ref | ref | 56 | ref | ref |
| High diversity | 142 | 63 | 1.00 (0.71,1.42) | 1.01 (0.70,1.45) | 52 | 0.96 (0.66,1.40) | 0.97 (0.65,1.44) |
| Abbreviations: Faith PD, Faith phylogenetic diversity. | | | | | | | |
| ^†^HRs were adjusted for age, sex, smoking history, BMI before treatment, cancer stage, treatment pattern, alcohol consumption, the number of missing or repaired teeth, sequence running number, residential community and season of saliva sampling. | | | | | | | |
|  |  |  |  |  |  |  |  |

### Table G

**Table G. Hazard ratios of tertiled PCs from RPCA on mortality using Cox regression**

|  | | | All-cause HRs | |  | NPC-specific HRs | |
| --- | --- | --- | --- | --- | --- | --- | --- |
| PCs^†^ | Cases   (n=427) | Deaths   (n=193) | Crude | Adjusted^*^ | Deaths of NPC   (n=167) | Crude | Adjusted^*^ |
| **PC1** |  |  |  |  |  |  |  |
| tertile 1 | 143 | 58 | ref | ref | 48 | ref | ref |
| tertile 2 | 142 | 65 | 1.21 (0.85,1.72) | 0.96 (0.64,1.44) | 58 | 1.30 (0.89,1.90) | 1.05 (0.69,1.61) |
| tertile 3 | 142 | 70 | 1.32 (0.93,1.86) | 1.10 (0.74,1.64) | 61 | 1.39 (0.95,2.02) | 1.14 (0.74,1.75) |
| **PC2** |  |  |  |  |  |  |  |
| tertile 1 | 143 | 68 | ref | ref | 62 | ref | ref |
| tertile 2 | 142 | 63 | 0.88 (0.63,1.24) | 0.75 (0.52,1.09) | 54 | 0.83 (0.58,1.20) | 0.75 (0.51,1.11) |
| tertile 3 | 142 | 62 | 0.91 (0.64,1.28) | 0.81 (0.54,1.21) | 51 | 0.82 (0.56,1.18) | 0.78 (0.51,1.21) |
| **PC3** |  |  |  |  |  |  |  |
| tertile 1 | 143 | 75 | ref | ref | 68 | ref | ref |
| tertile 2 | 142 | 59 | **0.71 (0.50,1.00)** | **0.62 (0.42,0.92)** | 50 | **0.67 (0.46,0.96)** | **0.65 (0.43,0.98)** |
| tertile 3 | 142 | 59 | 0.72 (0.51,1.01) | **0.57 (0.37,0.86)** | 49 | **0.66 (0.46,0.95)** | **0.59 (0.37,0.92)** |
| Abbreviation: RPCA, robust Aitchison principal-component analysis.  ^†^ The feature loadings were transformed by multiplied by -1. | | | | | | | |
| ^*^Adjusted for age at diagnosis, sex, sequencing running number, tobacco use, the number of missing or repaired tooth, cancer stage, BMI before treatments, alcohol consumption, diagnosis calendar year, treatment pattern, saliva sampling season, residential community and Faith phylogenetic diversity. | | | | | | | |
|  |  |  |  |  |  |  |  |

### Figure A


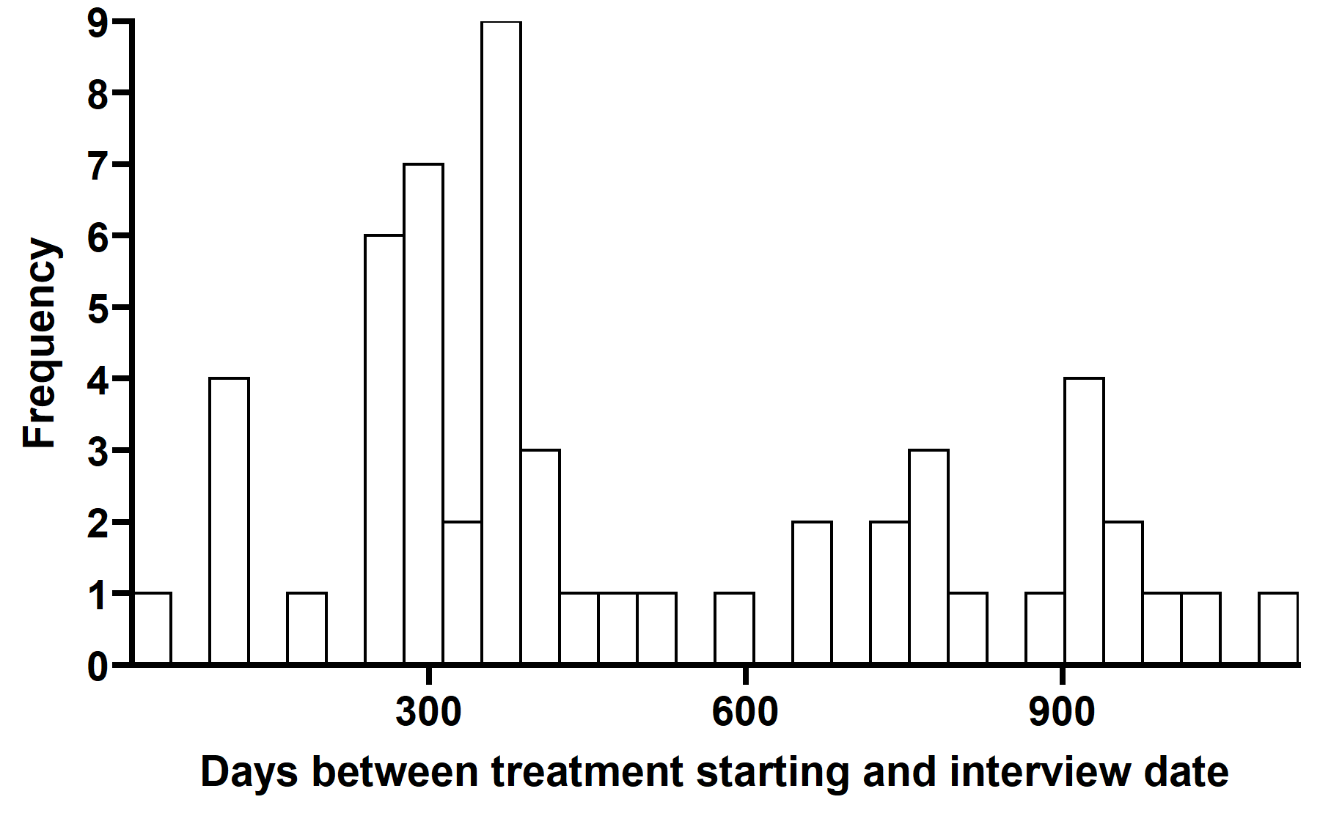


Figure A: Histogram showing the interval days between treatment starting and interview date of 55 cases whose saliva samples were collected during or after treatment.
