# Supplementary material for: Influence of Pre-treatment Saliva Microbial Diversity and Composition on Nasopharyngeal Carcinoma Prognosis
Source: Front Cell Infect Microbiol. 2022 Mar 22;12:831409. doi: 10.3389/fcimb.2022.831409 (PMC8981580; doi:10.3389/fcimb.2022.831409)
Supplement: Supplementary file 1 [file DataSheet_1.zip › Supplementary_OtherSupportingMaterials/Supplementary_frontiers_20220313.docx]

# Supplement for paper ‘Influence of pre-treatment saliva microbial diversity and composition on nasopharyngeal carcinoma prognosis’

Contents

[Supplement for paper ‘Influence of pre-treatment saliva microbial diversity and composition on nasopharyngeal carcinoma prognosis’ 1](#_Toc93495125)

[Supplement methods 3](#_Toc93495126)

[Method S1: Saliva collection 3](#_Toc93495127)

[Method S2: Saliva sample processing 3](#_Toc93495128)

[Method S3: Code to generate Robust Aitchison principal-component analysis (RPCA) 4](#_Toc93495129)

[Missing values 5](#_Toc93495130)

[Figure supplement 6](#_Toc93495131)

[Figure S1 6](#_Toc93495132)

[Figure S2 7](#_Toc93495133)

[Figure S3 9](#_Toc93495134)

[Figure S4 10](#_Toc93495135)

[Figure S5 11](#_Toc93495136)

[Figure S6 12](#_Toc93495137)

[Figure S7 13](#_Toc93495138)

[Table supplement 14](#_Toc93495139)

[Table S1 14](#_Toc93495140)

[Table S2 15](#_Toc93495141)

[Table S3 16](#_Toc93495142)

[Table S4 17](#_Toc93495143)

[Table S5 18](#_Toc93495144)

[References 20](#_Toc93495145)

# Supplement methods

## Method S1: Saliva collection

1). Make sure that the specimen provider has no diet and no chewing gum within 30 minutes of collecting saliva.

2). Use the prepared centrifuge tube containing 2mL lysis buffer (50 mM Tris, PH=8; 50 mM EDTA; 50nM Sucrose; 100mM NaCl and 1% SDS), and then open the lid each time the saliva is spilt out, and close immediately after spitting.

3). During collecting saliva, first stimulates to increase saliva production, such as using the tip of the tongue against the upper jaw, helping the provider massage both cheeks, and telling the provider not to swallow saliva. After about 2 minutes, there are approximately 5 ml of saliva in the mouth.

4). Ask the participants to place saliva in the mouth and gently brush their cheeks for about 10 seconds.

5). Open the centrifugal tube cap, bow your head, open your mouth, so that saliva flows naturally along the lower lip.

6). When the centrifuge tube is full of 4 mL (i.e. when about 2 mL of saliva is collected), close the lid. Mix well (flip up and down 8-10 times).

7). Saliva samples were stored at -20°C for up to three days and then transported to the laboratory and stored at −80°C.

## Method S2: Saliva sample processing

Before DNA extraction, lysozyme lysis was made of nuclease-free water, TE buffer and Triton X-100 (242:5:3). Each time, 23 samples and one autoclaved blank Eppendorf tube with nuclease-free water as a negative control were used for DNA extraction. 1mL saliva sample was mixed with 100µl lysozyme lysis buffer by vortex at low speed for 15 seconds; incubated at 37 °C for 60 min; added amount of 0.5-mm-diameter and 0.1-mm-diameter beads (BioSpec, Bartlesville, OK) to each sample, and blended for 10 min at top speed to disrupt the microbial cells physically. The clean liquid was transferred to a new 2ml Eppendorf tubes for each sample.

Further isolation and purification of the total DNA were completed as manufacturer’s instruction using TIANGEN TIANamp Blood DNA Kit. DNA concentration was measured Qubit fluorescence-based quantification system (Qubit 3.0 Fluorometer, Thermo Fisher Scientific, Wilmington, DE, USA).

The 16s rRNA amplicon library was amplified with 341F/805R primers CTACGGGNGGCWGCAG,GACTACHVGGGTATCTAATCC)^1^ ^2^. Samples were amplified with 20 cycles of a program with 30 seconds at 98°C for melting, 30 second at 60°C, and 30 seconds at 72°C. Next, samples were barcoded and cleaned up in a second PCR step^1^. DNA purity and volume were measured on an Agilent 2100 Bioanalyzer system and Real-time polymerase chain reaction. Sequencing was performed at Beijing Genome Institute on an Illumina MiSeq using a 2x300bp paired-end strategy.

**Quality controls for experiments:**

1). We ran DNA extraction, PCR and then sent them to BGI.

2). During DNA extraction, randomly selected 23 saliva samples and one negative control sample were treated as one batch for DNA extraction.

3). After DNA extraction, the concentration for 1 ml extracted saliva should be between 0 ng/µL to >600 ng/mL on qubit machine.

4). The sample quality was checked before sending them to the company. (1) The negative control was 0 ng/mL. (2) The concentration of samples should not be 0.

## Method S3: Code to generate Robust Aitchison principal-component analysis (RPCA)

RPCA was generated using *DEICODE* in QIIME2^3^. The code is shown below. It’s also deposited in [github](https://github.com/xinyueandtianliangle/Oral-microbiome-and-NPC-prognosis/blob/main/RPCA_QIIME2.Rmd).

**Code to generate RPCA**

Input dataset is feature table with raw count (raw_feature_table.qza). Output datasets are ordination file (ordination.qza) and distance metrics (distance.qza).

qiime deicode rpca \

    --i-table raw_feature_table.qza \

    --p-min-feature-count 0 \

    --p-min-sample-count 6500 \

    --o-biplot ordination.qza \

--o-distance-matrix distance.qza

**Code to generate biplot**

Input datasets are ordination file (ordination.qza), metadata file (metadata.tsv) and taxonomic file (taxa.tsv). Output file is qzv file (biplot_DEICODE.qzv) which can be visualized in EMPEROR.

qiime emperor biplot \

--i-biplot ordination.qza \

--m-sample-metadata-file metadata.tsv \

--m-feature-metadata-file taxa.tsv \

--o-visualization biplot_DEICODE.qzv \

--p-number-of-features 8

## Missing values

Baseline covariates were complete for 97.4% (482/495) subjects. The number of missingness for each covariate was mild: cancer stage (12/482), treatment pattern (12/482) and BMI before treatment (13/482). We used a complete case analysis in this manuscript considering the aspects below: 1) when the number of missingness is rare (< 5%), we could regard it as missing completely at random (MCAR) and use listwise deletion^4^. 2) we used Little’s Test^5^ to test whether the missing pattern was MCAR using *r* package (*BaylorEdPsych, 0.5 version*) and it wasn’t against MCAR assumption. 3) The test to find beta diversity contributors, *Adonis,* cannot deal with missing values.

# Figure supplement

## Figure S1

*
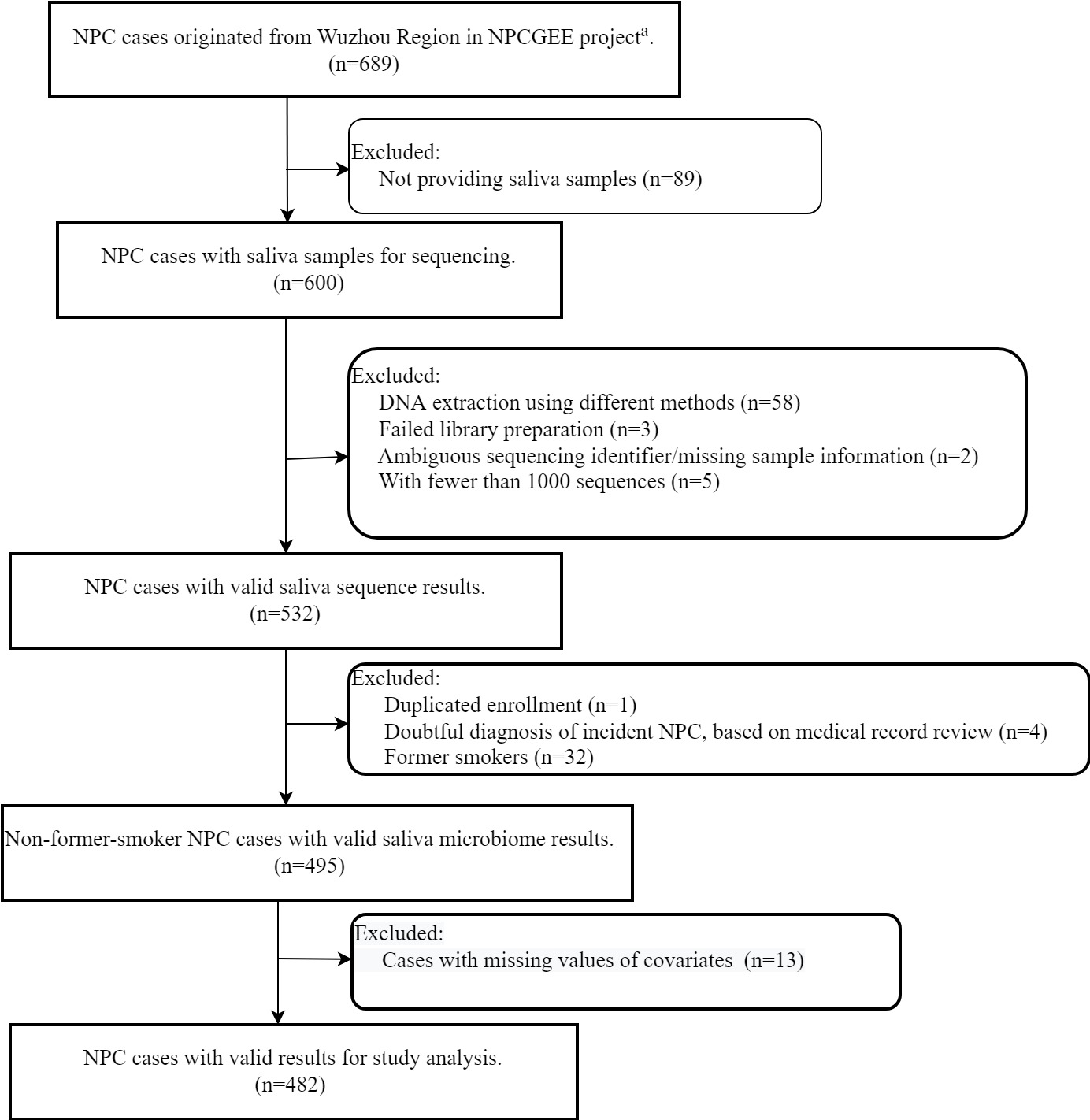
*

**Figure S1. Flow chart of study population.**

NPC, nasopharyngeal carcinoma.

^a^NPCGEE, Gene-environment EBV Interactions in the Etiology of NPC^6^.

## Figure S2

*
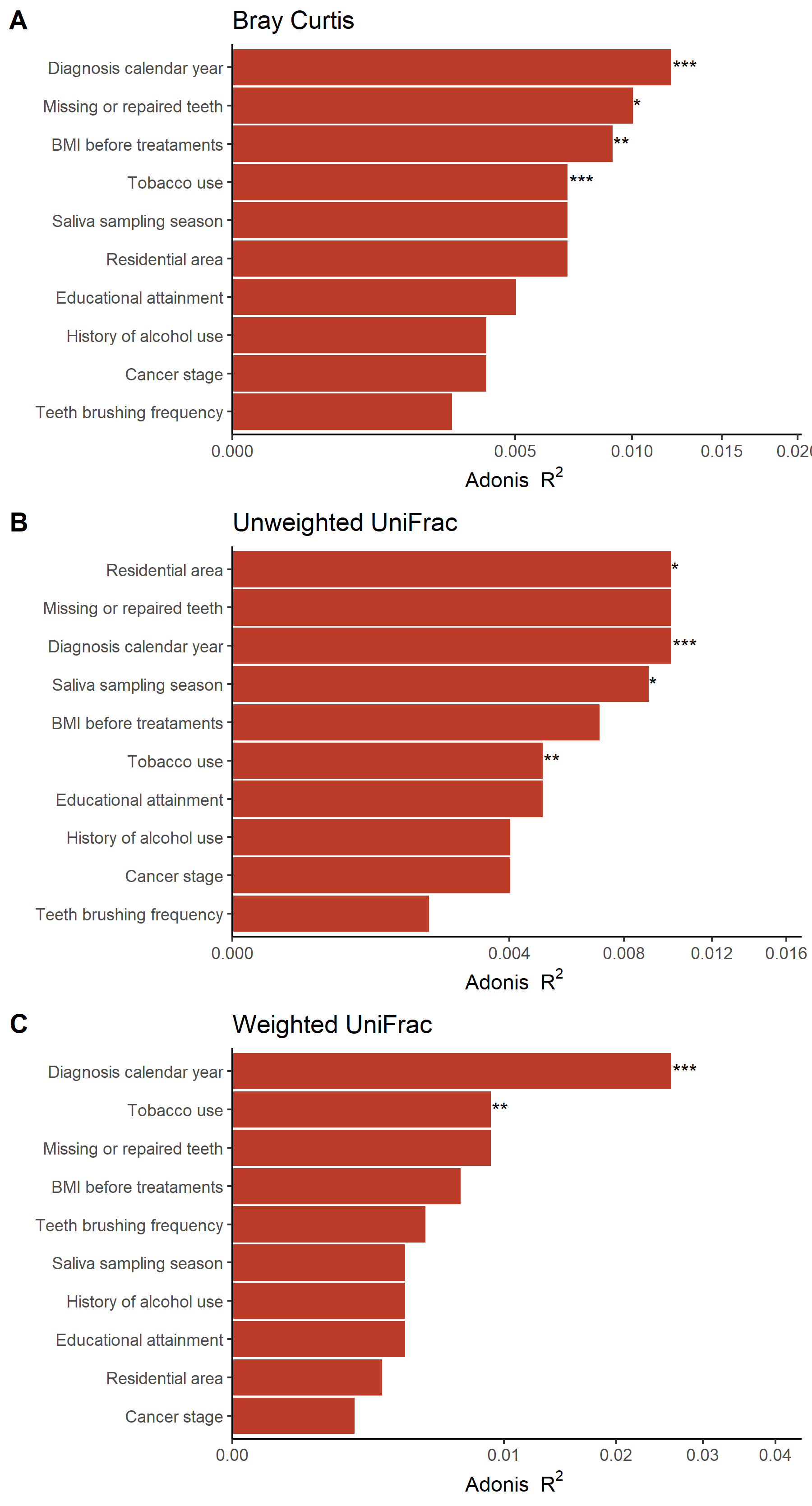
*

**Figure S2**: **Adonis testing (permutation=999) with a model adjusted for age, sex and sequencing number for beta diversity matrix.**

R^2^ was estimated variance explained by the variable in the model.

Smoking history, diagnosis calendar year, BMI before treatment, residential community and saliva sampling season were significant beta diversity contributors.

**,* FDR-adjusted *p* <0.05; **, FDR-adjusted *p* <0.01; ***, FDR-adjusted *p* <.001.

## Figure S3

| *A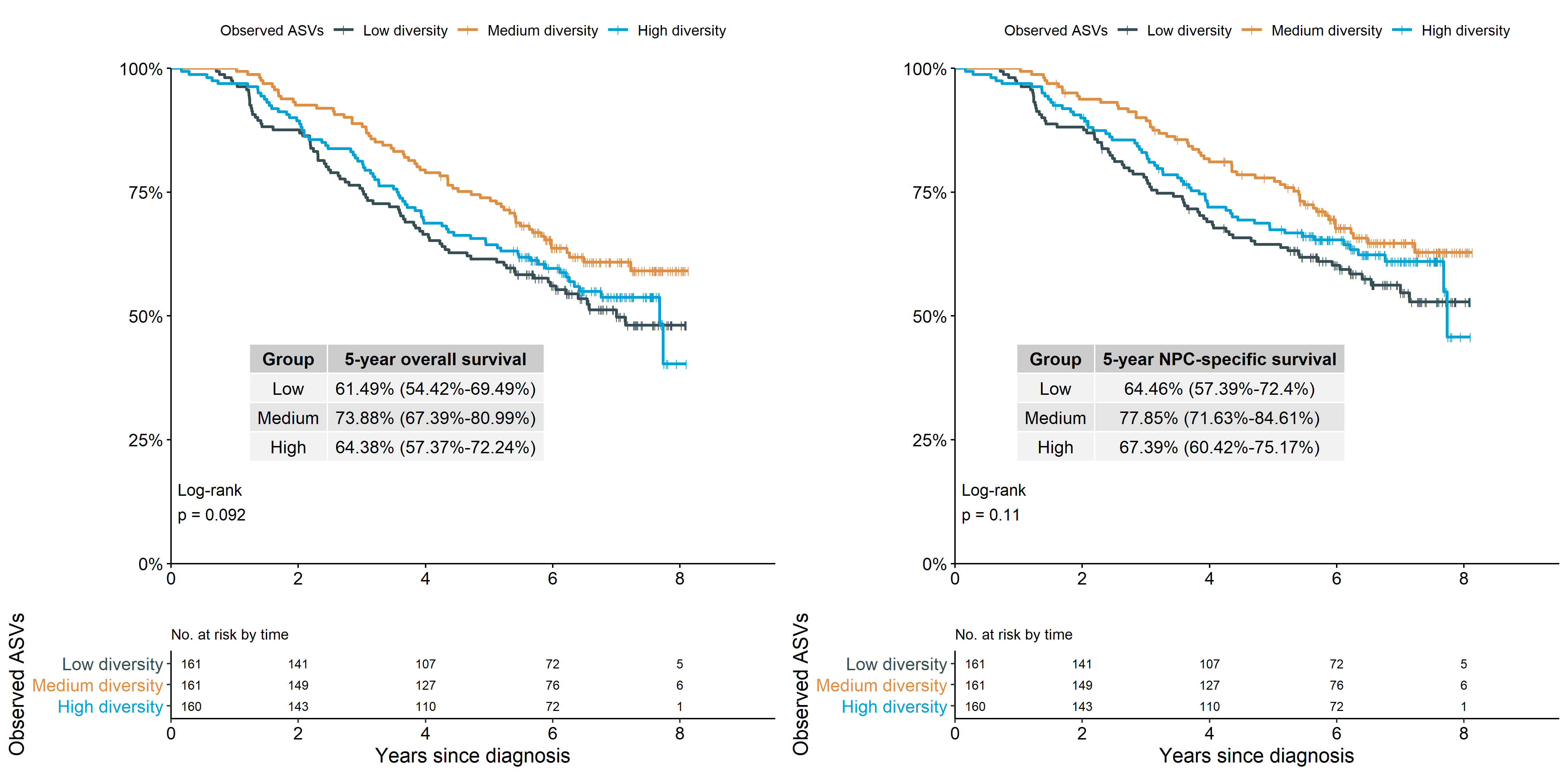* |
| --- |
| *B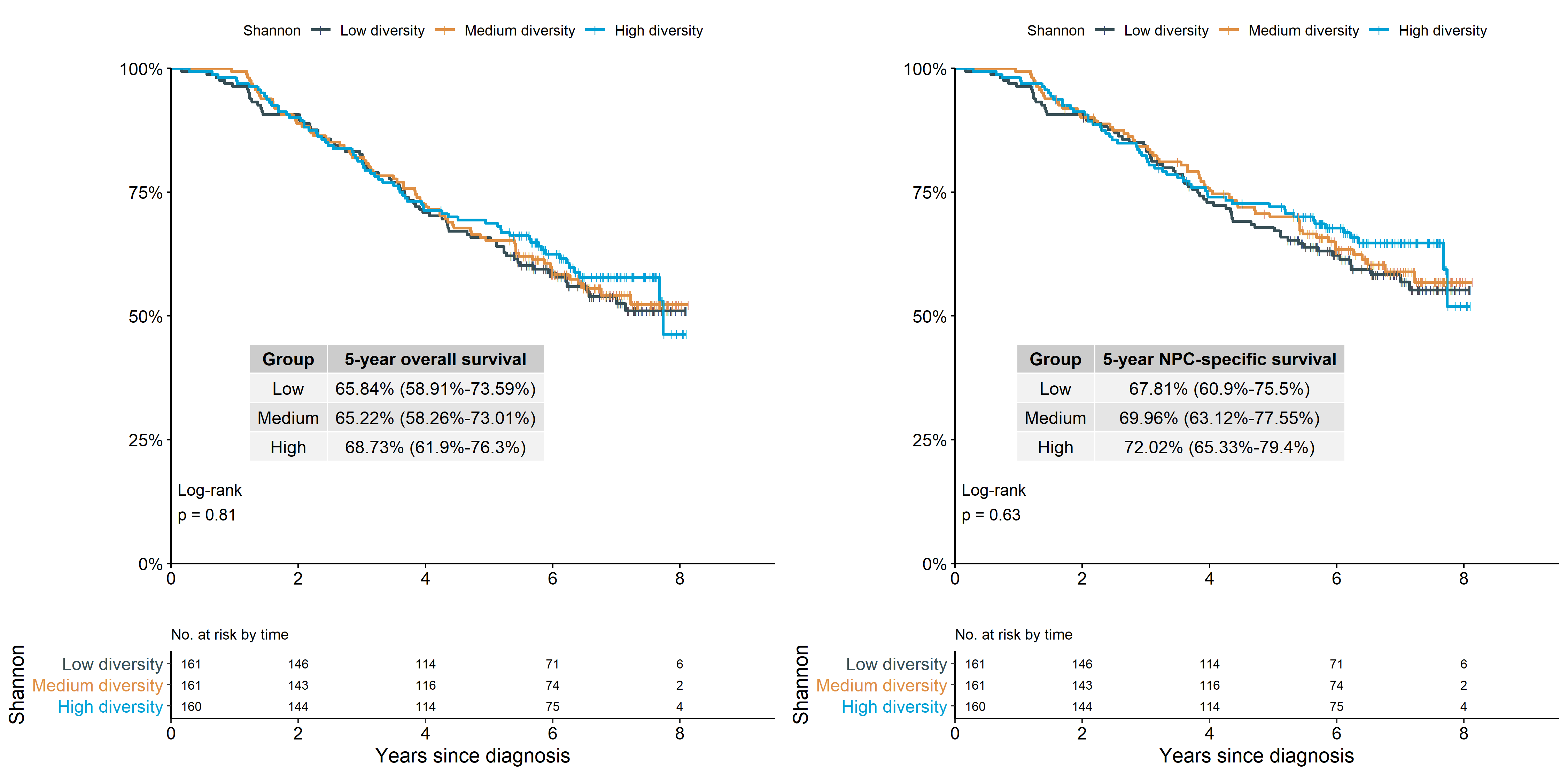* |

**Figure S3: Survival proportion of NPC cases by alpha diversity (Observed ASVs (Panel A) and Shannon diversity (Panel B))**

Abbreviations: NPC, nasopharyngeal carcinoma; ASV, amplicon sequences variants.

## Figure S4

| *A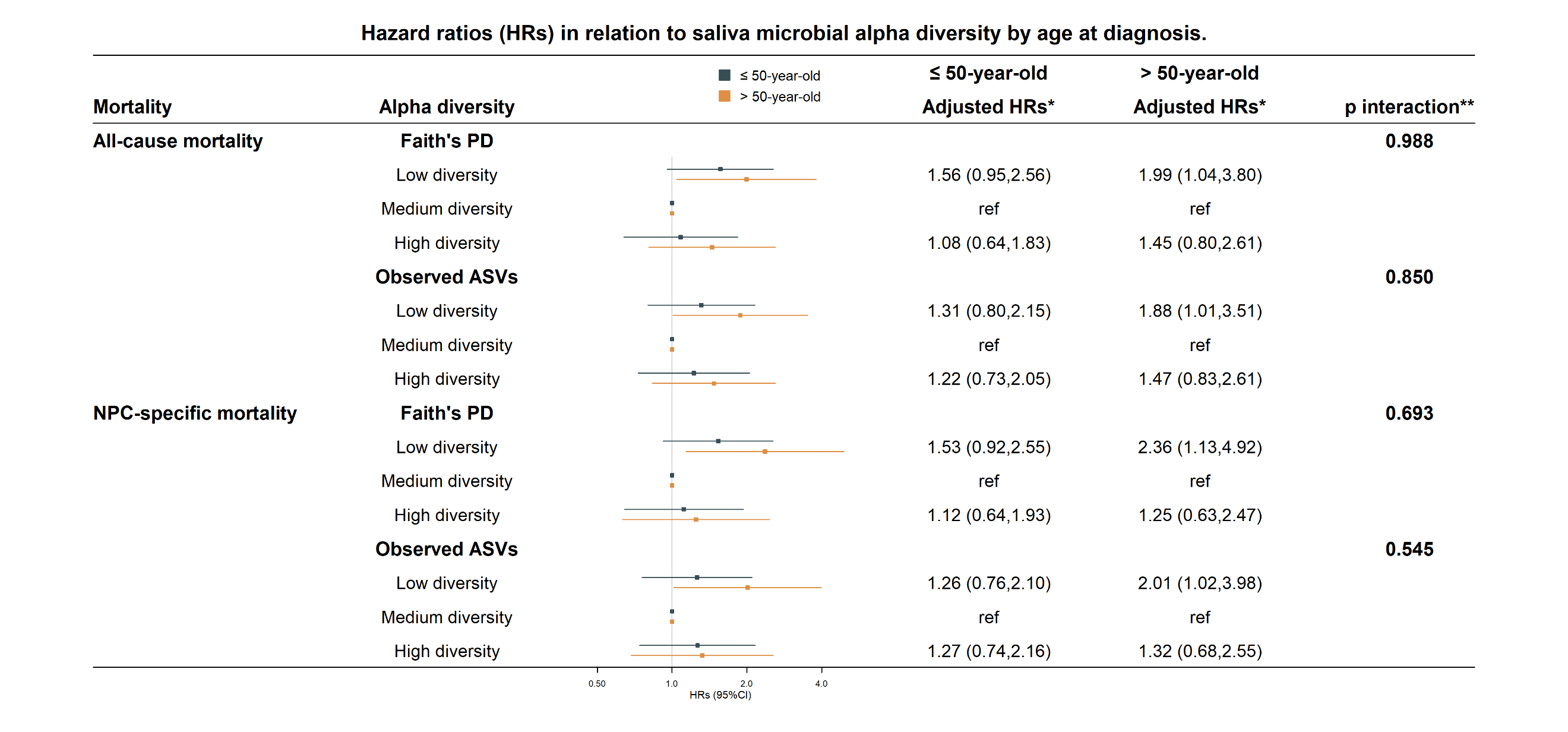* |
| --- |
| *B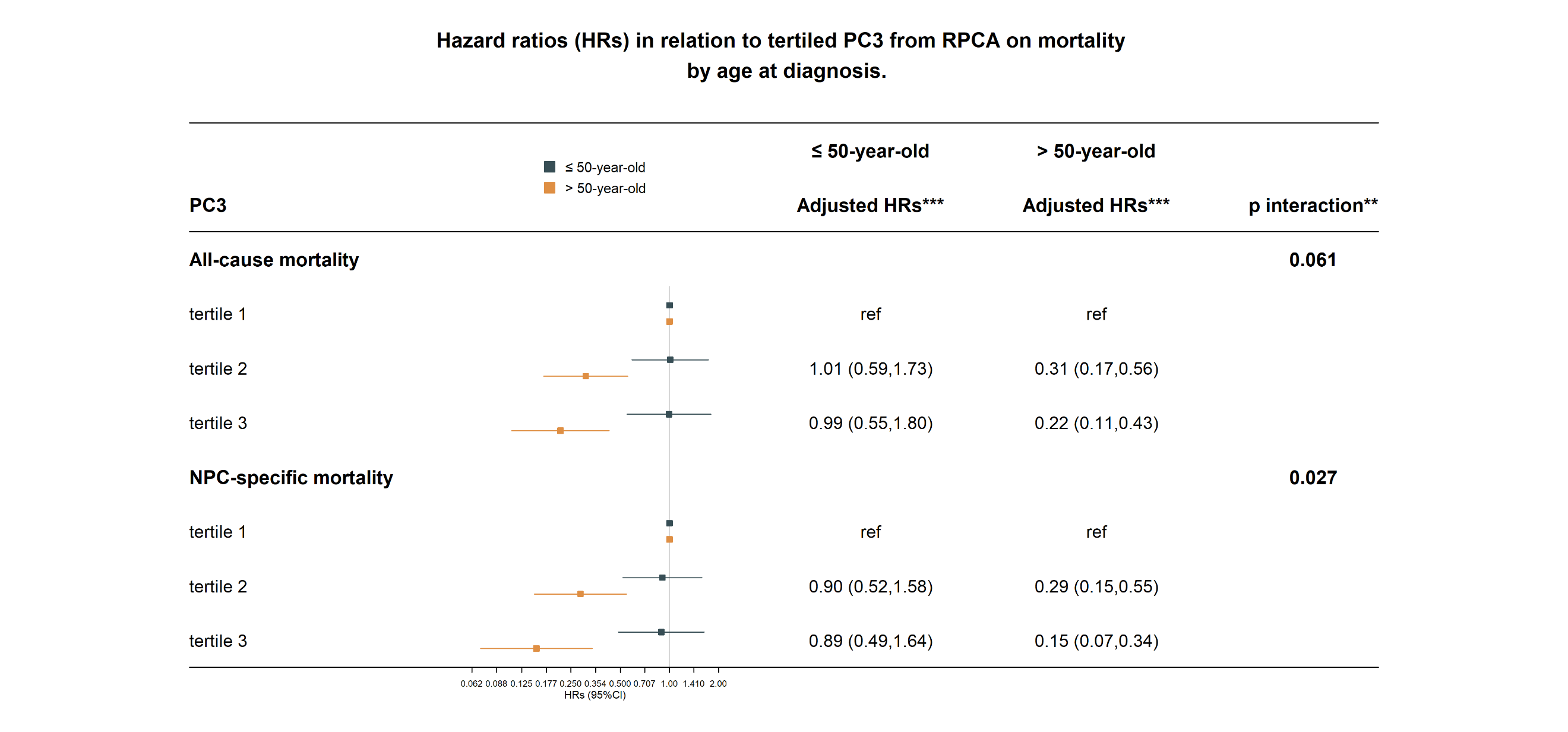* |

**Figure S4: Hazard ratios (HRs) in relation to saliva microbial alpha diversity (Faith’s PD and observed ASVs) stratified by age at diagnosis (A), and tertiled PC3 from RPCA stratified by age at diagnosis (B).**

Abbreviations: Faith’s PD, Faith’s phylogenetic diversity; PCoA, principal-coordinates analysis; RPCA, robust Aitchison principal-component analysis; NPC, nasopharyngeal carcinoma.

*Adjusted for age at diagnosis, sex, smoking history, BMI before treatment, cancer stage, treatment pattern, alcohol history, the number of missing or repaired teeth, sequence running number, residential community and saliva sampling season.

** Values of p interaction was generated to compare nested models with and without interaction term by likelihood ratio test.

*** Adjusted for age at diagnosis, sex, smoking history, diagnosis calendar year, BMI before treatment, cancer stage, treatment pattern, alcohol history, the number of missing or repaired teeth, sequence running number, residential community, saliva sampling season and Faith’s phylogenetic diversity.

## Figure S5


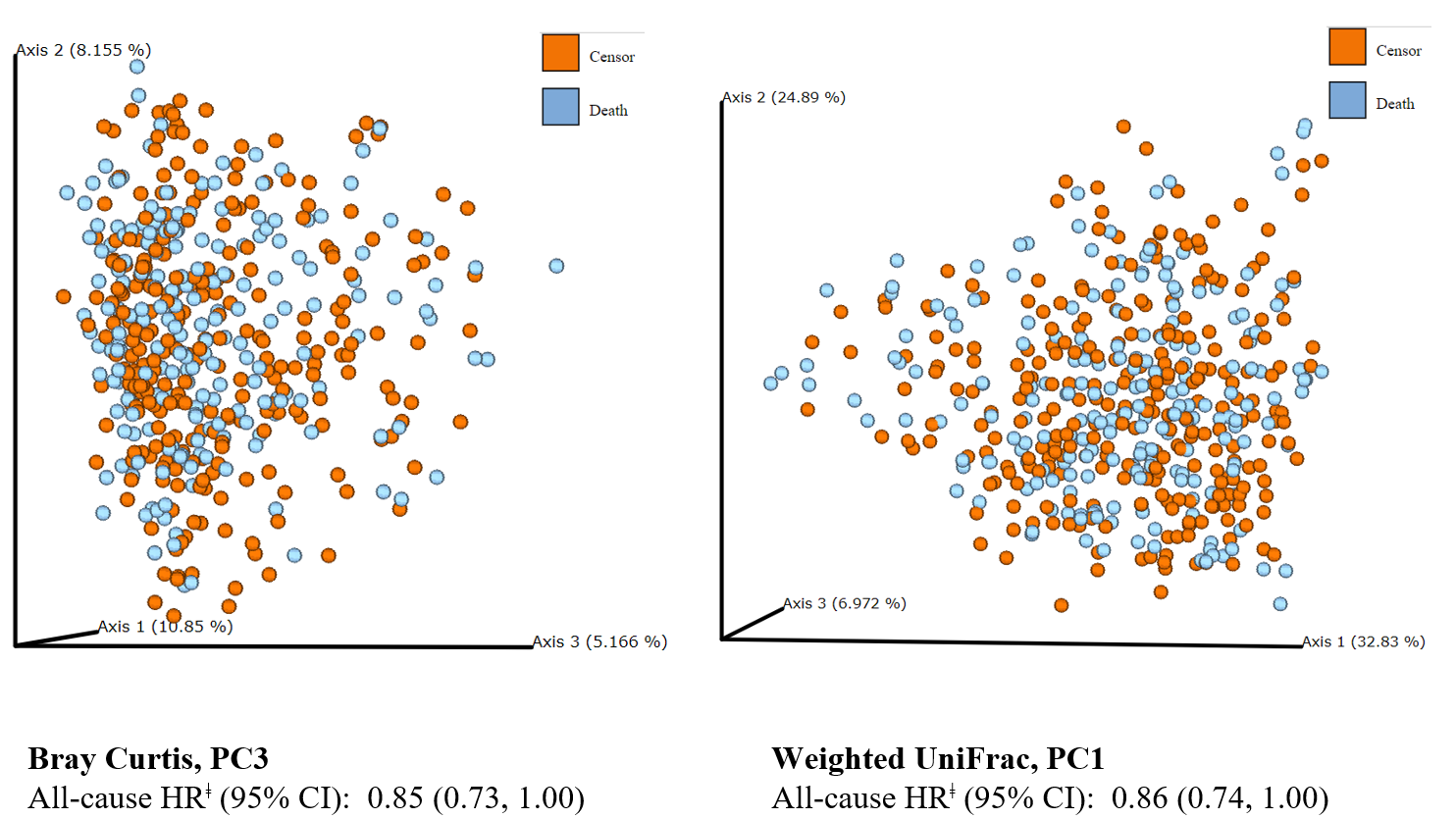


**Figure S5: PCoA of Bray Curtis distance (Left) and weighted UniFrac distance (Right).**

PC3 of Bray Curtis PCoA (Left) were significantly associated with all-cause mortality in Cox model with an adjusted HR 0.85 (95% CI, 0.73 - 1.00, *p* =0.046). PC1 of weighted UniFrac PCoA (Right) was marginally significantly associated with all-cause mortality in Cox model with an adjusted HR 0.85 (95% CI, 0.73 - 1.00, *p* =0.078). Axis1, axis2 and axis3 were equal to PC1, PC2 and PC3. The axes were labelled with the variation proportion that PCs explain. Sample loadings PC3 were z-normalized in Cox models.

Abbreviations: PCoA, principal-coordinates analysis; NPC, nasopharyngeal carcinoma.

^ǂ^ HRs were adjusted for age at diagnosis, sex, sequencing running number, tobacco use, the number of missing or repaired tooth, cancer stage, treatment pattern, saliva sampling season, BMI before treatments, alcohol use, diagnosis calendar year, residential community and Faith’s PD. PC3 were z-normalized.

## Figure S6

| 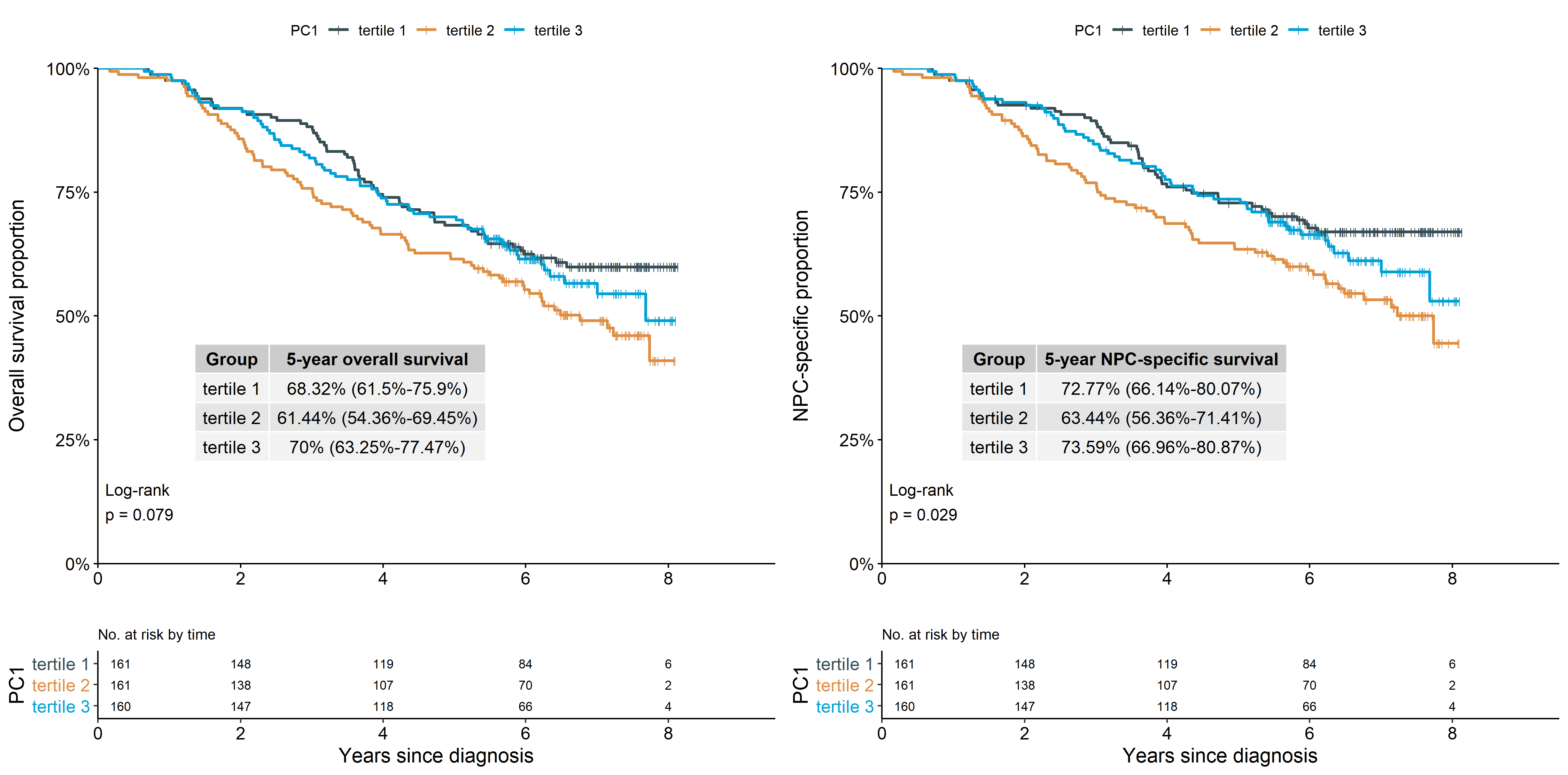 |
| --- |
| 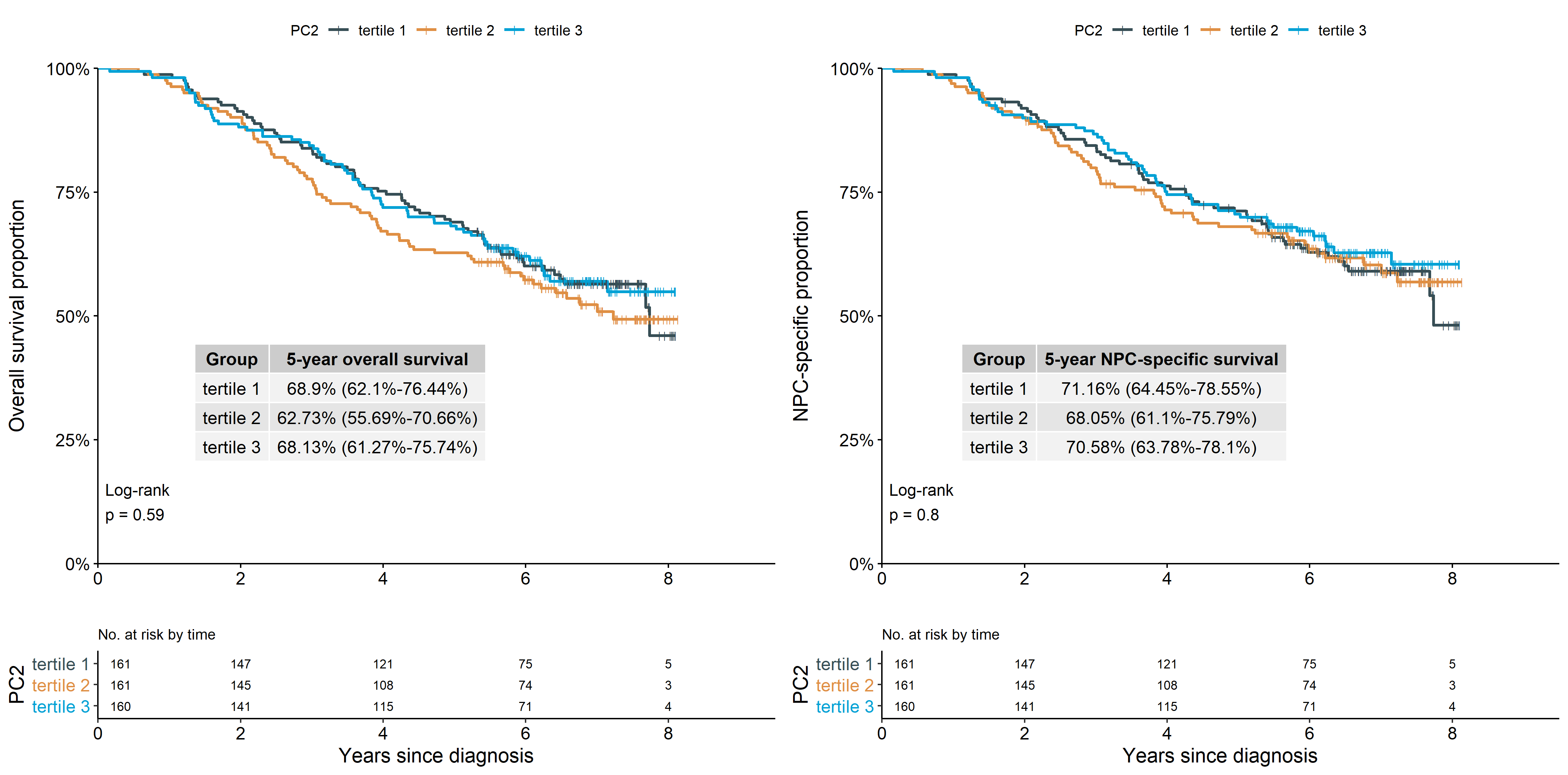 |

**Figure S6: Kaplan Meier curves of overall survival and NPC-specific survival proportion between tertiled PC1 and PC2 groups generated from RPCA.**

Abbreviations: RPCA, robust Aitchison principal-component analysis.

## Figure S7


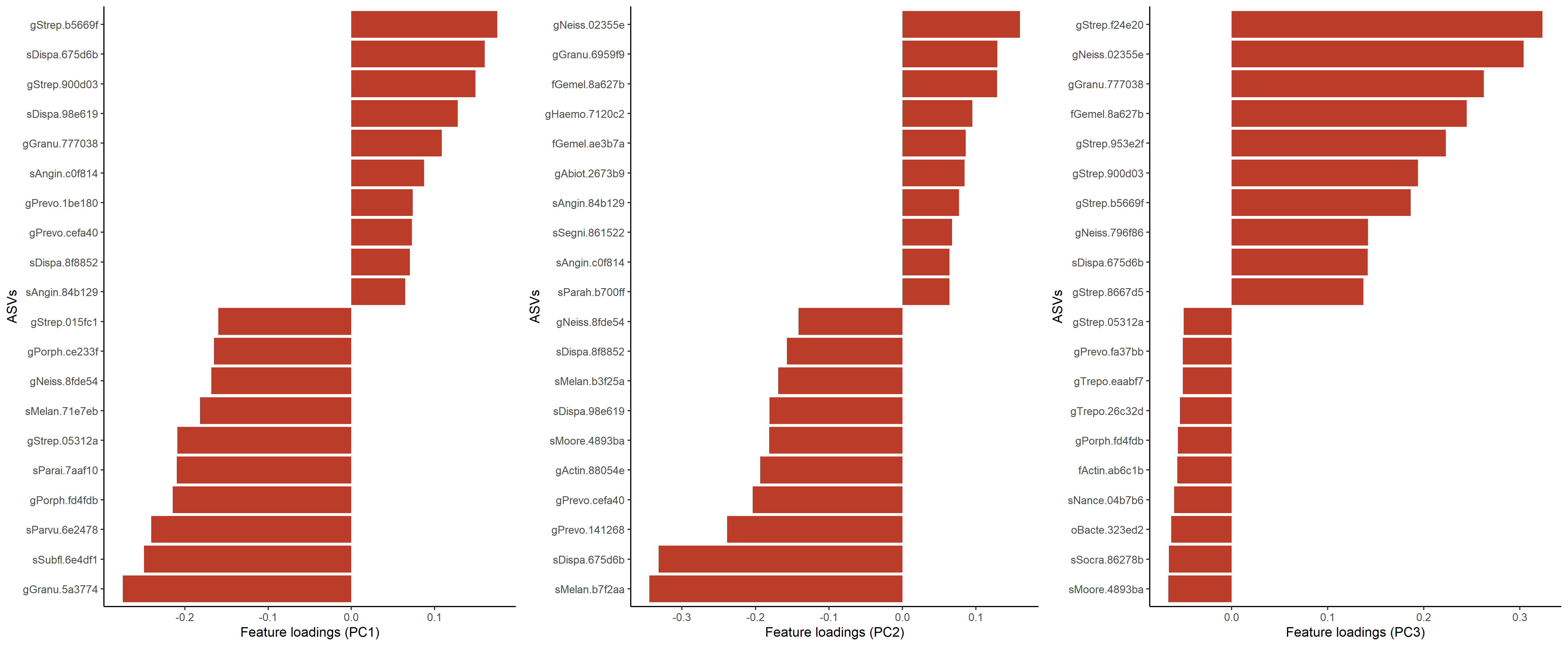


**Figure S7: Top and bottom 10 ASVs with highest and lowest feature loadings of PC1 (left), PC2 (middle) and PC3 (right) from RPCA.**

Abbreviations: RPCA, robust Aitchison principal-component analysis;

# Table supplement

## Table S1

**Table S1. Kruskal-Wallis rank sum test between potential alpha diversity contributors and alpha diversity metrics**

|  | **Faith’s PD** | | **Observed ASVs** | | **Shannon diversity** | |
| --- | --- | --- | --- | --- | --- | --- |
| **Characteristics** | **Statistics** | ***p*** | **Statistics** | ***p*** | **Statistics** | ***p*** |
| Residential community | 7.06 | 0.070 | 9.03 | **0.029** | 4.53 | 0.209 |
| Educational attainment | 1.37 | 0.503 | 0.75 | 0.686 | 3.43 | 0.180 |
| Tobacco use | 0.12 | 0.732 | 0.35 | 0.554 | 0.00 | 0.980 |
| Diagnosis calendar year | 1.50 | 0.473 | 0.52 | 0.771 | 1.40 | 0.497 |
| Tooth brushing frequency | 0.04 | 0.836 | 0.08 | 0.780 | 3.02 | 0.082 |
| Missing or repaired teeth | 2.84 | 0.585 | 2.20 | 0.700 | 2.36 | 0.670 |
| Cancer stage | 1.08 | 0.582 | 1.29 | 0.526 | 0.53 | 0.765 |
| BMI before treatments | 3.12 | 0.373 | 1.98 | 0.576 | 2.76 | 0.430 |
| History of alcohol use | 1.53 | 0.465 | 2.63 | 0.268 | 2.33 | 0.313 |
| Saliva sampling season | 7.53 | 0.057 | 3.74 | 0.291 | 1.66 | 0.645 |
| Abbreviations: Faith’s PD, Faith’s phylogenetic diversity. | | | | | | |

## Table S2

| Table S2. Analysis of deviance table for nested with versus without top three PCoA coordinates separately   \|  \| \| \| All-cause mortality \| \| NPC-specific mortality \| \| \| --- \| --- \| --- \| --- \| --- \| --- \| --- \| \| Beta diversity \| PCs \| Proportion   of variation % \| *p^a^* \| *p* (alpha)^b^ \| *p*^a^ \| *p* (alpha)^b^ \| \| Bray curtis \| PC1 \| 10.85 \| 0.456 \| 0.468 \| 0.681 \| 0.562 \| \|  \| PC2 \| 8.15 \| 0.544 \| 0.698 \| 0.427 \| 0.531 \| \|  \| PC3 \| 5.17 \| 0.061 \| **0.041** \| 0.132 \| 0.178 \| \| Unweighted UniFrac \| PC1 \| 20.98 \| 0.104 \| 0.442 \| 0.049 \| 0.545 \| \|  \| PC2 \| 5.99 \| 0.681 \| 0.683 \| 0.984 \| 0.586 \| \|  \| PC3 \| 3.99 \| 0.382 \| 0.430 \| 0.416 \| 0.492 \| \| Weighted UniFrac \| PC1 \| 32.83 \| 0.102 \| **0.053** \| 0.171 \| 0.186 \| \|  \| PC2 \| 24.89 \| 0.434 \| 0.407 \| 0.940 \| 0.580 \| \|  \| PC3 \| 6.97 \| 0.294 \| 0.530 \| 0.205 \| 0.486 \| \| *p* values were generated using likelihood ratio tests to compare nested models.   Abbreviations: PCs, PCoA coordinates; Faith’s PD, Faith’s phylogenetic diversity. \| \| \| \| \| \| \| \| ^a^Adjusted for age, sex, sequencing running number, tobacco use, the number of missing or repaired tooth, cancer stage, treatment pattern, saliva sampling season, BMI before treatments, alcohol use, diagnosis calendar year and residential community. \| \| \| \| \| \| \| \| ^b^ a adjusted plus Faith’s PD. \| \| \| \| \| \| \| \|  \| \| \| \| \| \| \| \|  \| \| \| \| \| \| \| \| |
| --- | --- | --- | --- | --- | --- | --- | --- | --- | --- | --- | --- | --- | --- | --- | --- | --- | --- | --- | --- | --- | --- | --- | --- | --- | --- | --- | --- | --- | --- | --- | --- | --- | --- | --- | --- | --- | --- | --- | --- | --- | --- | --- | --- | --- | --- | --- | --- | --- | --- | --- | --- | --- | --- | --- | --- | --- | --- | --- | --- | --- | --- | --- | --- | --- | --- | --- | --- | --- | --- | --- | --- | --- | --- | --- | --- | --- | --- | --- | --- | --- | --- | --- | --- | --- | --- | --- | --- | --- | --- | --- | --- | --- | --- | --- | --- | --- | --- | --- | --- | --- | --- | --- | --- | --- | --- | --- | --- | --- | --- | --- | --- | --- |

## Table S3

Table S3. Hazard ratios (HRs) for mortality of NPC cases in relation to numeric RPCA components, Cox regression models

|  | All-cause HRs | | NPC-specific HRs | |
| --- | --- | --- | --- | --- |
| PCs | Crude | Adjusted^a^ | Crude | Adjusted^a^ |
| PC1 | 1.05 (0.91,1.20) | 1.10 (0.94,1.30) | 1.04 (0.90,1.20) | 1.08 (0.90,1.28) |
| PC2 | 0.95 (0.83,1.08) | 0.96 (0.81,1.13) | 0.92 (0.79,1.06) | 0.93 (0.78,1.11) |
| PC3 | **0.85 (0.74,0.97)** | **0.72 (0.61,0.85)** | **0.80 (0.69,0.93)** | **0.71 (0.60,0.85)** |
| PCs were z-normalized.   Abbreviations: RPCA, robust Aitchison principal component analysis. | | | | |
| ^a^Adjusted for age, sex, sequencing running number, tobacco use, the number of missing or repaired tooth, cancer stage, BMI before treatments, alcohol use, diagnosis calendar year, treatment pattern, saliva sampling season, residential community and Faith’s phylogenetic diversity. | | | | |
|  |  |  |  |  |

## Table S4

Table S4. Hazard ratios (HRs) for mortality of NPC cases in relation to ALR-transformed features with the top 10 highest and the bottom 10 lowest feature loadings for PC3 from RPCA, Cox regression models

|  | All-cause | | | NPC-specific | | |
| --- | --- | --- | --- | --- | --- | --- |
| ASVs^a^ | HRs^b^ | p | FDR | HRs^b^ | p | FDR |
| gStrep.f24e20 | 0.92 (0.79,1.07) | 0.273 | 0.511 | 1.05 (0.84,1.32) | 0.660 | 0.741 |
| gNeiss.02355e | 0.97 (0.92,1.01) | 0.156 | 0.511 | 0.97 (0.92,1.02) | 0.191 | 0.681 |
| gGranu.777038 | 0.98 (0.93,1.03) | 0.437 | 0.513 | 0.99 (0.93,1.05) | 0.704 | 0.741 |
| fGemel.8a627b | 0.92 (0.85,1.00) | 0.042 | 0.415 | 0.94 (0.87,1.02) | 0.158 | 0.681 |
| gStrep.953e2f | 1.00 (0.94,1.06) | 0.911 | 0.958 | 1.02 (0.96,1.08) | 0.599 | 0.741 |
| gStrep.900d03 | 0.96 (0.91,1.01) | 0.142 | 0.511 | 0.96 (0.90,1.01) | 0.143 | 0.681 |
| gStrep.b5669f | 0.95 (0.89,1.02) | 0.160 | 0.511 | 0.94 (0.88,1.01) | 0.103 | 0.681 |
| gNeiss.796f86 | 0.97 (0.93,1.02) | 0.282 | 0.511 | 0.97 (0.91,1.02) | 0.206 | 0.681 |
| sDispa.675d6b | 1.02 (0.97,1.07) | 0.430 | 0.513 | 1.02 (0.97,1.07) | 0.412 | 0.741 |
| gStrep.8667d5 | 0.96 (0.89,1.03) | 0.253 | 0.511 | 0.96 (0.89,1.04) | 0.350 | 0.741 |
| **gStrep.05312a** | 1.06 (1.01,1.11) | **0.018** | 0.368 | 1.05 (1.00,1.10) | 0.068 | 0.681 |
| gPrevo.fa37bb | 1.09 (0.93,1.27) | 0.284 | 0.511 | 1.08 (0.91,1.27) | 0.393 | 0.741 |
| gTrepo.eaabf7 | 1.08 (0.92,1.26) | 0.361 | 0.513 | 1.05 (0.88,1.24) | 0.608 | 0.741 |
| gTrepo.26c32d | 1.06 (0.91,1.24) | 0.448 | 0.513 | 1.04 (0.88,1.23) | 0.671 | 0.741 |
| gPorph.fd4fdb | 1.03 (0.97,1.09) | 0.305 | 0.511 | 1.02 (0.97,1.08) | 0.451 | 0.741 |
| fActin.ab6c1b | 1.06 (0.91,1.24) | 0.461 | 0.513 | 1.02 (0.86,1.21) | 0.783 | 0.783 |
| sNance.04b7b6 | 1.00 (0.94,1.07) | 0.961 | 0.961 | 0.98 (0.92,1.05) | 0.594 | 0.741 |
| oBacte.323ed2 | 1.05 (0.93,1.18) | 0.440 | 0.513 | 1.03 (0.90,1.18) | 0.638 | 0.741 |
| sSocra.86278b | 1.08 (0.94,1.24) | 0.306 | 0.511 | 1.05 (0.91,1.23) | 0.495 | 0.741 |
| sMoore.4893ba | 1.06 (0.97,1.16) | 0.166 | 0.511 | 1.06 (0.96,1.17) | 0.238 | 0.681 |
| Abbreviation: RPCA, robust Aitchison principal-component analysis; ALR, additive-log-ratio. | | | | | | |
| ^a^ Features with the top 10 highest and the bottom 10 lowest feature loadings for PC3 from RPCA. The abundance was additive-log-ratio (ALR) transformed. The reference frame was features with the top 10 highest feature loadings for PC3 from RPCA. | | | | | | |
| ^b^Adjusted for age, sex, sequencing running number, tobacco use, the number of missing or repaired tooth, cancer stage, BMI before treatments, alcohol use, diagnosis calendar year, treatment pattern, saliva sampling season and residential community. | | | | | | |
|  |  |  |  |  |  |  |

## Table S5

**Table S5. Referred ASVs in this analysis and their taxonomic assignment and MD5 hash**

| ASVs ID | Taxon | MD5 hash |
| --- | --- | --- |
| gGranu.777038 | k__Bacteria; p__Firmicutes; c__Bacilli; o__Lactobacillales; f__Carnobacteriaceae; g__Granulicatella; s__ | 7770387e217bc9cbc12dcb87a16f779d |
| gStrep.900d03 | k__Bacteria; p__Firmicutes; c__Bacilli; o__Lactobacillales; f__Streptococcaceae; g__Streptococcus; s__ | 900d037057012d2c1f6932e79447d6e5 |
| gStrep.b5669f | k__Bacteria; p__Firmicutes; c__Bacilli; o__Lactobacillales; f__Streptococcaceae; g__Streptococcus; s__ | b5669fcddd91a072c53111ddbe3ebb4e |
| sAngin.c0f814 | k__Bacteria; p__Firmicutes; c__Bacilli; o__Lactobacillales; f__Streptococcaceae; g__Streptococcus; s__anginosus | c0f8141ce37fee3000d4975c19521a63 |
| sAngin.84b129 | k__Bacteria; p__Firmicutes; c__Bacilli; o__Lactobacillales; f__Streptococcaceae; g__Streptococcus; s__anginosus | 84b129f1b0ad2f90cee394e6bb8b4dbf |
| gPrevo.cefa40 | k__Bacteria; p__Bacteroidetes; c__Bacteroidia; o__Bacteroidales; f__Prevotellaceae; g__Prevotella; s__ | cefa404b8e5183bf720ad80fa4c60536 |
| gPrevo.1be180 | k__Bacteria; p__Bacteroidetes; c__Bacteroidia; o__Bacteroidales; f__Prevotellaceae; g__Prevotella; s__ | 1be180033a1595929d88040e13a5e05d |
| sDispa.675d6b | k__Bacteria; p__Firmicutes; c__Clostridia; o__Clostridiales; f__Veillonellaceae; g__Veillonella; s__dispar | 675d6bb61a724d1662fbdd2ec8e2fdb5 |
| sDispa.8f8852 | k__Bacteria; p__Firmicutes; c__Clostridia; o__Clostridiales; f__Veillonellaceae; g__Veillonella; s__dispar | 8f8852458a0ee511c0195dce33d0c2bb |
| sDispa.98e619 | k__Bacteria; p__Firmicutes; c__Clostridia; o__Clostridiales; f__Veillonellaceae; g__Veillonella; s__dispar | 98e6193e3661ea8ee8ef0fd328872fcc |
| gGranu.5a3774 | k__Bacteria; p__Firmicutes; c__Bacilli; o__Lactobacillales; f__Carnobacteriaceae; g__Granulicatella; s__ | 5a377461bd57a8f49193086a97e6a6d0 |
| gStrep.015fc1 | k__Bacteria; p__Firmicutes; c__Bacilli; o__Lactobacillales; f__Streptococcaceae; g__Streptococcus; s__ | 015fc1a6061b24f7c72d68cf4eb65728 |
| gStrep.05312a | k__Bacteria; p__Firmicutes; c__Bacilli; o__Lactobacillales; f__Streptococcaceae; g__Streptococcus; s__ | 05312af8bfc19ad043bba8eef08fc993 |
| sMelan.71e7eb | k__Bacteria; p__Bacteroidetes; c__Bacteroidia; o__Bacteroidales; f__Prevotellaceae; g__Prevotella; s__melaninogenica | 71e7ebc90d440c7133fc5f2ad5d4a8e4 |
| gPorph.fd4fdb | k__Bacteria; p__Bacteroidetes; c__Bacteroidia; o__Bacteroidales; f__Porphyromonadaceae; g__Porphyromonas; s__ | fd4fdba1ce5a4ed6fd0737d58bf50f39 |
| gPorph.ce233f | k__Bacteria; p__Bacteroidetes; c__Bacteroidia; o__Bacteroidales; f__Porphyromonadaceae; g__Porphyromonas; s__ | ce233f21bd523923b70dafe43e9d1ab8 |
| sParai.7aaf10 | k__Bacteria; p__Proteobacteria; c__Gammaproteobacteria; o__Pasteurellales; f__Pasteurellaceae; g__Haemophilus; s__parainfluenzae | 7aaf10574cf708d59b1bea48318533de |
| sParvu.6e2478 | k__Bacteria; p__Firmicutes; c__Clostridia; o__Clostridiales; f__Veillonellaceae; g__Veillonella; s__parvula | 6e2478236b76509338fa471879295c61 |
| gNeiss.8fde54 | k__Bacteria; p__Proteobacteria; c__Betaproteobacteria; o__Neisseriales; f__Neisseriaceae; g__Neisseria | 8fde5464ccdd05d3fea84dfe6b2157ff |
| sSubfl.6e4df1 | k__Bacteria; p__Proteobacteria; c__Betaproteobacteria; o__Neisseriales; f__Neisseriaceae; g__Neisseria; s__subflava | 6e4df1779ce4ddac1d34d3aad1b1147a |
| gGranu.6959f9 | k__Bacteria; p__Firmicutes; c__Bacilli; o__Lactobacillales; f__Carnobacteriaceae; g__Granulicatella; s__ | 6959f96a526b7a50dee1e59e3bdd5f03 |
| gAbiot.2673b9 | k__Bacteria; p__Firmicutes; c__Bacilli; o__Lactobacillales; f__Aerococcaceae; g__Abiotrophia; s__ | 2673b9d00108b71efa27f54e616b6efd |
| fGemel.8a627b | k__Bacteria; p__Firmicutes; c__Bacilli; o__Gemellales; f__Gemellaceae; g__; s__ | 8a627befd417e0e1518533f8bd426b6f |
| fGemel.ae3b7a | k__Bacteria; p__Firmicutes; c__Bacilli; o__Gemellales; f__Gemellaceae | ae3b7a4378e8a2ee26662c700b5110ed |
| sParah.b700ff | k__Bacteria; p__Proteobacteria; c__Gammaproteobacteria; o__Pasteurellales; f__Pasteurellaceae; g__Actinobacillus; s__parahaemolyticus | b700ff92ca00c39f7a9a9f1dff823b37 |
| sSegni.861522 | k__Bacteria; p__Proteobacteria; c__Gammaproteobacteria; o__Pasteurellales; f__Pasteurellaceae; g__Aggregatibacter; s__segnis | 8615227e1f44f73d218c07608f2bf31a |
| gHaemo.7120c2 | k__Bacteria; p__Proteobacteria; c__Gammaproteobacteria; o__Pasteurellales; f__Pasteurellaceae; g__Haemophilus | 7120c2d159a61477dfb5d177c00aa8c0 |
| gNeiss.02355e | k__Bacteria; p__Proteobacteria; c__Betaproteobacteria; o__Neisseriales; f__Neisseriaceae; g__Neisseria; s__ | 02355e9120de68a8e20abf582469a057 |
| sMoore.4893ba | k__Bacteria; p__Firmicutes; c__Erysipelotrichi; o__Erysipelotrichales; f__Erysipelotrichaceae; g__Bulleidia; s__moorei | 4893bace34757913be61cdc1408e2201 |
| sMelan.b7f2aa | k__Bacteria; p__Bacteroidetes; c__Bacteroidia; o__Bacteroidales; f__Prevotellaceae; g__Prevotella; s__melaninogenica | b7f2aabaf0870e02656a317fbf473538 |
| gPrevo.141268 | k__Bacteria; p__Bacteroidetes; c__Bacteroidia; o__Bacteroidales; f__[Paraprevotellaceae]; g__[Prevotella]; s__ | 141268533d1a794604a2ec293076c2fc |
| sMelan.b3f25a | k__Bacteria; p__Bacteroidetes; c__Bacteroidia; o__Bacteroidales; f__Prevotellaceae; g__Prevotella; s__melaninogenica | b3f25a3f3f9d8cf64fc0581fab0f5875 |
| gActin.88054e | k__Bacteria; p__Actinobacteria; c__Actinobacteria; o__Actinomycetales; f__Actinomycetaceae; g__Actinomyces; s__ | 88054ec4fd56a26d9a396648e6a25e9e |
| gStrep.8667d5 | k__Bacteria; p__Firmicutes; c__Bacilli; o__Lactobacillales; f__Streptococcaceae; g__Streptococcus; s__ | 8667d5eb1b26e8d4ee26c60ddabe7bde |
| gStrep.f24e20 | k__Bacteria; p__Firmicutes; c__Bacilli; o__Lactobacillales; f__Streptococcaceae; g__Streptococcus | f24e20c0a6014a8e36ecff03211f7a42 |
| gStrep.953e2f | k__Bacteria; p__Firmicutes; c__Bacilli; o__Lactobacillales; f__Streptococcaceae; g__Streptococcus; s__ | 953e2f8c5f6d08f71431e8d44797edfe |
| gNeiss.796f86 | k__Bacteria; p__Proteobacteria; c__Betaproteobacteria; o__Neisseriales; f__Neisseriaceae; g__Neisseria; s__ | 796f86de542fde7bc276e6b75dc226d9 |
| gTrepo.eaabf7 | k__Bacteria; p__Spirochaetes; c__Spirochaetes; o__Spirochaetales; f__Spirochaetaceae; g__Treponema; s__ | eaabf7ff022e340b373a65e8ddab7e33 |
| gTrepo.26c32d | k__Bacteria; p__Spirochaetes; c__Spirochaetes; o__Spirochaetales; f__Spirochaetaceae; g__Treponema; s__ | 26c32d630935c23e88dd25fae1f88590 |
| sSocra.86278b | k__Bacteria; p__Spirochaetes; c__Spirochaetes; o__Spirochaetales; f__Spirochaetaceae; g__Treponema; s__socranskii | 86278b2975bd8c4e3a33917e6685e112 |
| sNance.04b7b6 | k__Bacteria; p__Bacteroidetes; c__Bacteroidia; o__Bacteroidales; f__Prevotellaceae; g__Prevotella; s__nanceiensis | 04b7b60c5501f192eb97c168882cd617 |
| gPrevo.fa37bb | k__Bacteria; p__Bacteroidetes; c__Bacteroidia; o__Bacteroidales; f__Prevotellaceae; g__Prevotella; s__ | fa37bbe7aa3ab1839588f55738e6392f |
| oBacte.323ed2 | k__Bacteria; p__Bacteroidetes; c__Bacteroidia; o__Bacteroidales | 323ed2d507255a104adabae1450d3910 |
| fActin.ab6c1b | k__Bacteria; p__Actinobacteria; c__Actinobacteria; o__Actinomycetales; f__Actinomycetaceae; g__; s__ | ab6c1b0183f635f790ef534dec11ed7b |

|  |
| --- |

|  |
| --- |

# References

1. Hugerth LW, Wefer HA, Lundin S, et al. DegePrime, a program for degenerate primer design for broad-taxonomic-range PCR in microbial ecology studies. *Appl Environ Microbiol* 2014;80(16):5116-23. doi: 10.1128/aem.01403-14 [published Online First: 2014/06/15]

2. Rognes T, Flouri T, Nichols B, et al. VSEARCH: a versatile open source tool for metagenomics. *PeerJ* 2016;4:e2584. doi: 10.7717/peerj.2584 [published Online First: 2016/10/27]

3. Martino C, Morton JT, Marotz CA, et al. A Novel Sparse Compositional Technique Reveals Microbial Perturbations. *mSystems* 2019;4(1) doi: 10.1128/mSystems.00016-19 [published Online First: 2019/02/26]

4. Review of the Methods for Handling Missing Data in Longitudinal Data Analysis; 2011.

5. Little RJA. A Test of Missing Completely at Random for Multivariate Data with Missing Values. *Journal of the American Statistical Association* 1988;83(404):1198-202. doi: 10.1080/01621459.1988.10478722

6. Ye W, Chang ET, Liu Z, et al. Development of a population-based cancer case-control study in southern china. *Oncotarget* 2017;8(50):87073-85. doi: 10.18632/oncotarget.19692 [published Online First: 2017/11/21]
